# Linear and Nonlinear Learning from Spectroelectrochemical Data: Interrogation of PLS and CNN Behavior Under Experimental Scarcity

**DOI:** 10.3390/molecules31162836

**Published:** 2026-08-14

**Authors:** Abderrahman Atifi

**Affiliations:** Energy and Environment Science & Technology, Idaho National Laboratory, Idaho Falls, ID 83415, USA; abderrahman.atifi@inl.gov

**Keywords:** spectroelectrochemistry, cyclic voltammetry, data linearity, machine learning, chemometrics

## Abstract

Spectroelectrochemistry (SEC) provides unique information-rich datasets by coupling molecular spectroscopic fingerprints with electrochemical activity. Despite its richness, direct machine-learning (ML) analysis of SEC data under realistic experimental constraints and scarcity remains unexplored. This work examines how much spectroscopically encoded electrochemical information can be learned from minimal SEC training data in a chemically reversible two-electron redox system, and how model choice interacts with limited experimental diversity across scan rate. Using purely experimental SEC datasets collected at four scan rates (2, 3, 5, and 7 mV/s), partial least squares (PLS) and convolutional neural networks (CNNs) regressors are evaluated under several SEC-level training, validation, and test configurations. Model performance is assessed across three targets of increasing physical complexity, including species concentrations, derivative cyclic voltabsorptometry (DCVA) current, and experimental cyclic voltammetry (CV) current. Under single-SEC training, both models achieve the expected near-quantitative concentration prediction (R^2^ ~0.99), while performance decreases for DCVA (R^2^ ~0.93) and most substantially for CV current (R^2^ ~0.78), reflecting the progressively weaker and more indirect encoding of these targets within the absorbance data. Introducing minimal experimental diversity with only two distinct training SEC datasets enables both PLS and CNN models to generalize strongly to an unseen third SEC dataset, achieving maximum CV R^2^ values approaching ~0.98 in the most favorable configurations. CNN models extend the apparent linear performance ceiling observed for PLS by capturing localized, scan-rate-conditioned nonlinear correlations between spectral evolution and the experimentally measured CV response, yielding improved waveform reconstruction and greater robustness to training SEC dataset selection. These results demonstrate that, within the present chemically reversible and spectroscopically well-resolved SEC system, high-fidelity prediction of electrochemical targets can be achieved without large datasets when limited but strategically selected electrochemical diversity is introduced. SEC dataset linearity is further shown to be target-dependent and becomes operationally meaningful only when scan-rate space is sufficiently sampled. More broadly, this work establishes a controlled framework for investigating ML-enabled SEC dataset analysis under experimentally scarce conditions and provides guidance for experimental design and calibration in low-data spectroelectrochemical settings.

## 1. Introduction

Spectroelectrochemistry is a long-established analytical platform, developed over more than half a century, that enables the simultaneous acquisition of molecular spectroscopic information and electrochemical activity [1,2]. In both fundamental and applied settings, ranging from electrochemical characterization and separations to chemical sensing, process monitoring, and nuclear fuel reprocessing, there is a strong need to rapidly build reliable calibration models for species quantification and redox-state diagnosis while minimizing experimental time and resource consumption [3,4,5]. In these contexts, traditional one-factor-at-a-time experimental strategies and simple bilinear models are poorly suited, as they scale inefficiently, fail to capture coupled physicochemical effects, and struggle to remain robust under changing operating conditions [6,7,8]. These limitations motivate multivariate and data-driven approaches that can leverage the intrinsic spectral-electrochemical complexity of SEC data and learn transferable structure from a small number of carefully designed experiments rather than from large and redundant datasets.

Machine learning (ML) is now routinely used across chemistry to uncover patterns in complex data, accelerate analysis workflows, map redox properties, and enable predictive models that are difficult to derive analytically [9,10,11]. In electrochemistry, most chemometric and ML efforts have focused on purely electrochemical measurements, such as cyclic voltammetry and impedance spectroscopy [12,13,14,15,16]. Whereas SEC datasets are higher-dimensional and structurally more complex than pure electrochemical signals. Each SEC experiment generates a coupled manifold spanning potential, wavelength, and electrochemical timescale, embedding spectral overlap of several redox-state populations, along with faradaic and non-faradaic electrochemical signals. Conventionally, the SEC spectral complexity has been addressed primarily through multi-step chemometric workflows, most commonly evolving factor analysis (EFA) and multivariate curve resolution, that aim to extract pure-component spectra and concentration profiles prior to mechanistic interpretation [3,4,17,18,19,20,21,22]. While these unsupervised approaches have enabled deep insight into complex redox systems, they also require careful preprocessing, expert intervention, and sequential analysis steps. Despite extensive use of chemometrics and ML for spectroscopy and electrochemistry separately, there are still no broadly adopted frameworks that learn directly from SEC data to relate spectroscopic evolution to electrochemical response in a unified and data-driven manner [23,24,25,26,27,28].

Our recent work began to explore this gap by examining whether deep learning could extract meaningful structure from SEC matrices when supported by a scalable training foundation [29]. Using a hybrid strategy that combined experimentally measured spectral components with physics-based simulations, we demonstrated that neural networks could simultaneously learn concentrations, derivative currents, and mechanistic parameters from hybrid SEC data. While this approach established proof of principle, it relied on large (~10 k SEC dataset units, mostly to span the electrochemical parameter space) synthetic datasets and focused primarily on processed targets, leaving open key questions about learning directly from limited, purely experimental SEC datasets and simultaneously measured electrochemical currents. By shifting the focus from synthetic data abundance to experimental scarcity, and from processed targets to experimentally measured electrochemical observables, this work examines fundamental questions regarding the learnability and effective linearity of spectroelectrochemical data. The scope of this work does not intend to establish new electrochemical interpretations of the anthraquinone (AQ) system, optimize spectroelectrochemical instrumentation, or delve further into thin-layer electrochemical theory.

Motivated by emerging electrochemical data science efforts, this study investigates how much electrochemical information can be recovered from minimal SEC training data and how model behavior depends on target complexity and experimental diversity. Recent methodological studies have emphasized that model generalization should be evaluated using chemically or experimentally structured train/test partitions rather than only random data splits [30]. Although comparable machine-learning studies on SEC datasets remain scarce, these developments provide an appropriate methodological framework for the present work. To isolate these relationships under controlled conditions, the analysis is intentionally restricted to a well-known, chemically reversible, and spectroscopically well-resolved anthraquinone system, in which Beer–Lambert behavior is approximately preserved, and spectral evolution remains experimentally stable. Model training is deliberately limited to a small number of purely experimental SEC datasets, placing learning in a low-rank regime defined by the scarcity of independent experiments rather than matrix dimensionality. Within this constrained setting, the work evaluates how linear and nonlinear models learn and transfer spectroscopic–electrochemical relationships across scan rate using experimentally distinct single-SEC and paired-SEC training configurations. Beyond benchmarking PLS and CNN, the principal novelty of this work is the establishment of a quantitative framework for evaluating the effective learnability and linearity of SEC datasets under experimentally scarce conditions. By progressively increasing target complexity from concentrations to DCVA and experimental CV current, the study identifies a hierarchy of learnability and demonstrates that experimental diversity, rather than model complexity alone, is the dominant factor governing robust transfer across independent SEC datasets. While these conclusions are demonstrated here for a relatively well-behaved redox system, the framework establishes a baseline for understanding how increasing chemical, kinetic, and spectroscopic complexity may influence learnability and the experimental diversity required in future SEC-driven machine-learning studies.

## 2. Results and Discussion

### 2.1. SEC DataFrame and Modeling Architecture

**Figure 1:**
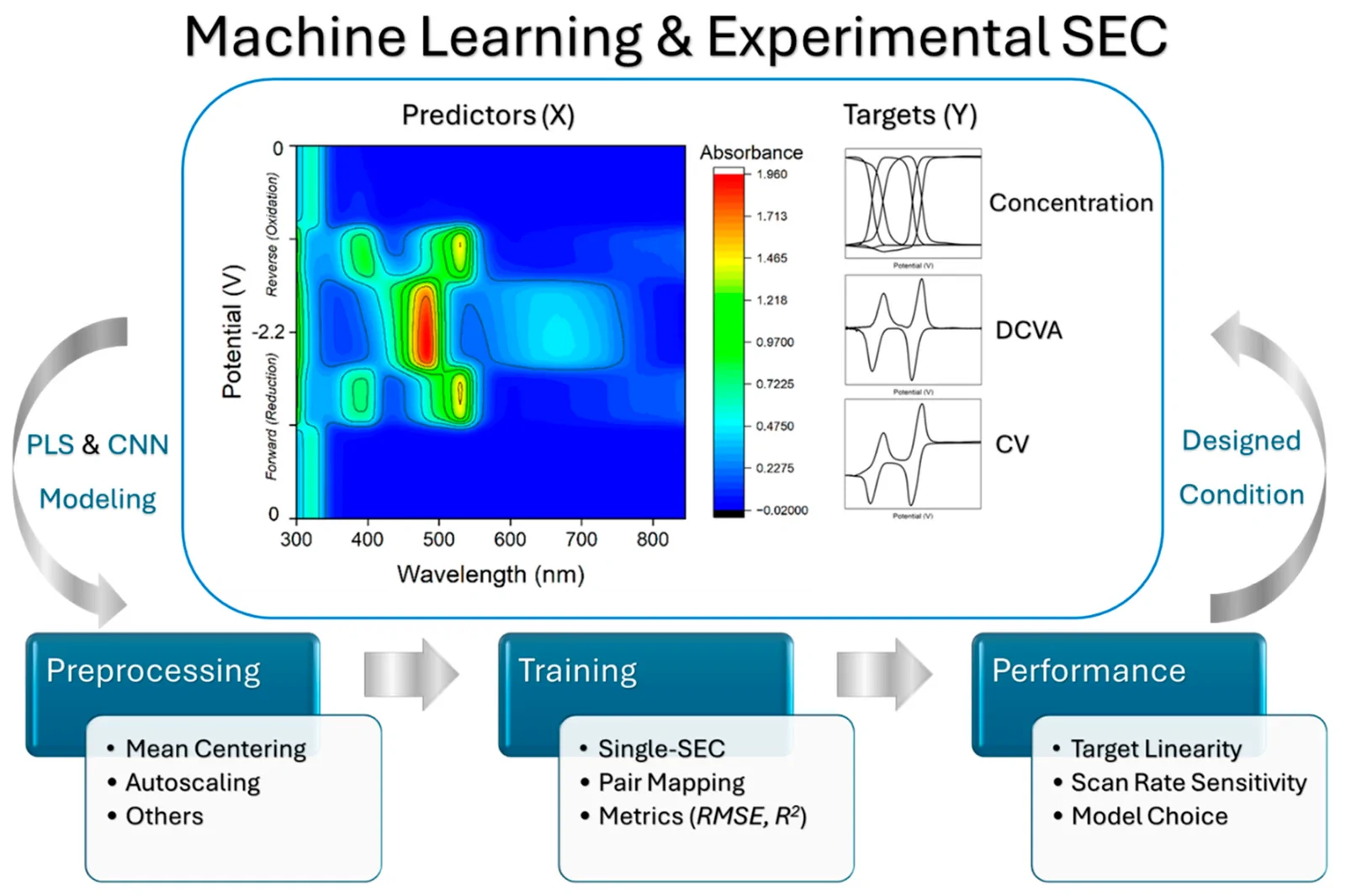
Schematic representation of the employed machine learning analysis of experimental SEC data in this work.

This study begins with training and evaluation using a single experimental SEC to establish a baseline for linear and nonlinear generalization across scan rate. This single-SEC regime (“regime” is used here to describe the computational training/validation/test configuration rather than an electrochemical diffusion regime) isolates the intrinsic structure of the spectroscopic-electrochemical mapping and reveals where linearity holds and where it begins to break down. The analysis is then extended to paired-SEC dataset mapping, where minimal but targeted experimental diversity is introduced, allowing assessment of how rapidly predictive performance improves when the learning problem is anchored at more than one electrochemical condition. The SEC DataFrame structuring and modeling workflow in this work are illustrated in Figure 1.

A representative experimental SEC dataset is embedded in Figure 1 as a two-dimensional contour map of absorbance versus wavelength and applied potential over a full forward and reverse cyclic voltammetry scan of the two-electron reduction of anthraquinone in aprotic media. The high absorbance symmetry between the forward and reverse scans is consistent with the chemical stability under the conditions studied, with clean potential-dependent spectral evolution and well-defined isosbestic behavior. Corresponding experimental CVs acquired at different scan rates are provided in Figure S2. Across all scan rates, the voltammograms exhibit minimal hysteresis and retain reversible wave shapes, consistent with high chemical stability and redox chemistry under these conditions.

These kinetically distinct SEC datasets provide a controlled platform for evaluating how linear and nonlinear models respond to increasing electrochemical complexity and scan-rate dependence. Each spectroelectrochemical dataset corresponds to a complete cyclic voltammetry experiment acquired at a fixed scan rate. Each structured SEC DataFrame is then represented by a coupled triplet (E^2001^, Abs^2001×1606^, Y^2001^). The predictor (X) matrix consists of wavelength-resolved absorbance (Abs) values, spanning 300–850 nm at each potential (E: from 0 to −2.2 V). The target vector Y corresponds to one of three quantities analyzed in this work: species concentration, DCVA current, or experimentally measured CV current. The concentration and DCVA targets were obtained from the previously reported EFA/DCVA deconvolution and Butler-Volmer derivation, where the full spectral decomposition methodology and concentration reconstruction procedures are detailed extensively [29]. Only four independent SEC datasets are employed in this study, acquired at scan rates of 2, 3, 5, and 7 mV/s.

In this work, two complementary model classes are evaluated for learning mappings from absorbance to electrochemical targets. PLS is a classical linear chemometric method widely used for spectroscopy, particularly suited for high-dimensional and collinear predictors [31]. PLS regression is implemented in a PLS1 formulation. The scaled predictor matrix and response vector are decomposed following Equation (1):

X = T P^T^; Y = T Q^T^ (1)

where T contains the latent variables (components), P are the X-loadings, and Q are the Y-loadings. For each configuration, the PLS analysis outputs included X-loadings and scores, Y-loadings, and SEC-level performance metrics (Test_R^2^ and Test_RMSE). X loadings describe spectroscopic contrasts that covary with electrochemical response, while Y loadings quantify how strongly each component (latent variable) contributes to the predicted target (CV current). Therefore, early components capture transferable, chemically meaningful structure (but not pure spectra), whereas higher components primarily model residual or scan rate-specific variance.

CNN model is introduced as a nonlinear deep learning counterpart designed to test whether the localized and structured nonlinearity in SEC data can be exploited beyond the limit of global linear projections [32]. Unlike PLS global latent subspace, CNN learns hierarchical feature representations through convolutional filters that operate locally along the potential axis, while integrating information across the spectral dimension. In this context, convolutional layers act as learned feature extractors that detect potential-localized spectral-electrochemical motifs, while subsequent fully connected layers combine these features to produce a scalar electrochemical prediction (Y value). To ensure a consistent comparison with PLS, the CNN model is trained using the same SEC-level split representation. Hyperparameters including the number of convolutional filters, kernel size, dense layer width, and regularization strength are optimized via a predefined sweep. Training is performed using the Adam optimizer with mean squared error loss, and early stopping based on validation loss is applied to prevent overfitting. The best-performing model is selected using minimum validation RMSE. The structure of the SEC DataFrame, including X and Y components, is illustrated in Figure 1. Prior to model training, several preprocessing methods were initially performed for the PLS model. The preprocessing screening results are summarized in Figure S1 and discussed in the Supplementary Materials.

### 2.2. PLS Performance with a Single Training SEC

**Figure 2:**
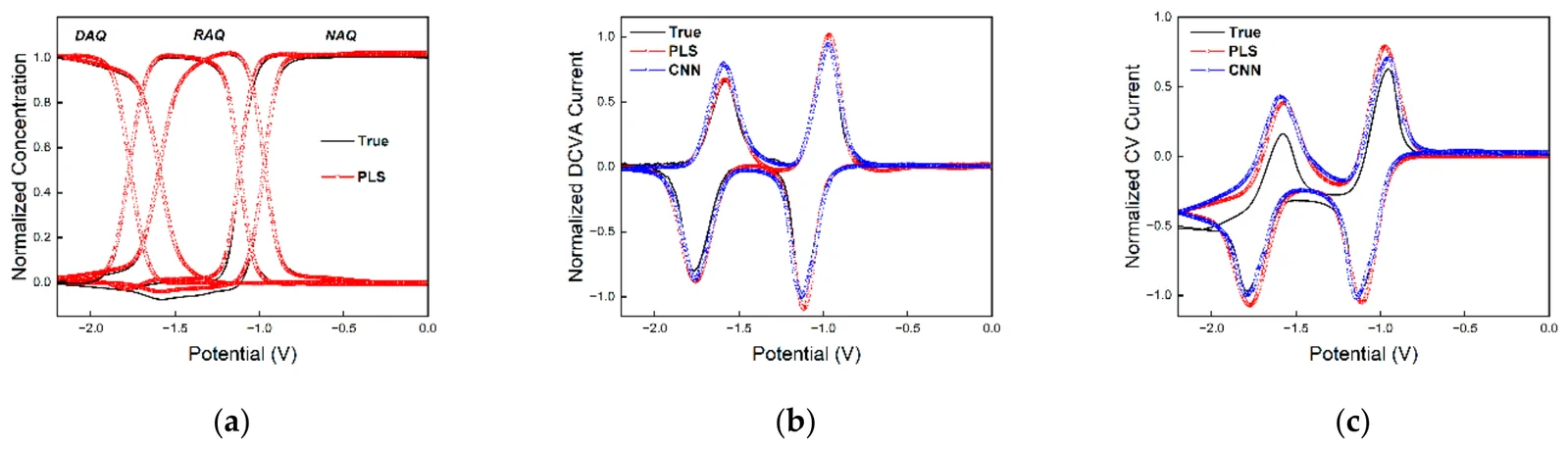
Performance of single-SEC dataset training of the 3 → 5 → 2 mV/s configuration, showing (**a**) PLS predictions versus true concentrations, (**b**) PLS and CNN predictions versus true DCVA current, and (**c**) PLS and CNN predictions versus experimental CV current.

PLS is first evaluated under single-SEC dataset training to establish the baseline capability of a linear latent-variable model under data sparsity. For a consistent discussion of obtained results, a representative configuration (3 → 5 → 2 mV/s) is illustrated: training at 3 mV/s, validation at 5 mV/s, and testing at 2 mV/s. This places the training SEC dataset at an intermediate scan rate while requiring extrapolation to both slower and faster scan-rate conditions. With concentrations as targets (Table S1), PLS achieves near-quantitative performance even with a single training SEC dataset, with Test_R^2^ values consistently near ≈0.99 across all examined single-SEC configurations and only negligible dependence on the choice of validation or test SEC dataset. For instance, in the representative 3 → 5 → 2 mV/s training-validation-test configuration (Figure 2a), Test_R^2^ values for concentration prediction exceed 0.985, with corresponding Test_RMSE values on the order of a few percent of the full concentration range. Test_RMSE does not vary across scan-rate combinations and remains close to the noise level. This behavior reflects the strong linearity imposed by the Beer–Lambert structure together with the orthogonal factor decomposition used previously to extract concentration profiles through Evolving Factor Analysis (EFA) [29]. Accordingly, the near-quantitative concentration predictions should not be interpreted as independent experimental validation, but rather as demonstrating the recoverability of an established EFA-derived representation using an independent regression framework. In the present study, the concentration target intentionally serves as a baseline representation of the SEC data, providing an upper bound for the learnability of the underlying absorbance–target relationship. Furthermore, although EFA-derived concentrations were used here to provide a well-established physics-based reference, concentration profiles could also be obtained independently through alternative analytical calibration methods. Because the dominant concentration variance is encoded directly within the absorbance manifold, concentration recoverability from SEC data is expected to remain high. However, the relationship is not entirely trivial: EFA concentrations arise from an unsupervised orthogonal decomposition of the absorbance space, whereas PLS constructs supervised latent variables that maximize covariance between absorbance and target spaces while preserving transferability across independent SEC datasets. In this regime, PLS operates near its best-case limit, where even a single SEC dataset spans a sufficiently informative absorbance–concentration subspace to support robust generalization across scan rates.

For DCVA current (Table S2), predictive performance drops to a moderate but still robust level under single-SEC dataset training, with Test_R^2^ values typically in the ~0.87–0.97 range. For example, in the 3 → 5 → 2 mV/s configuration (Figure 2b), DCVA’s highest Test_R^2^ value is ~0.97, with a corresponding Test_RMSE value about ~0.05. These values remain substantially good yet inferior to concentration prediction, indicating a meaningful loss of linear predictability. DCVA is obtained by differentiating concentration profiles with respect to potential (Butler-Volmer formalism), a process that introduces sensitivity to scan-rate-dependent changes in peak width, spacing, and curvature. Although absorbance retains much of the chemically relevant information, a single SEC dataset does not fully specify how these derivative features deform when scan rate changes. As a result, PLS begins to lose fidelity when forced to extrapolate to an unseen electrochemical condition.

The most pronounced limitations of single-SEC dataset PLS modeling emerge in the prediction of the experimental CV current (Figure 2c). Test_R^2^ value was about ~0.77 on average, with Test_RMSE values often in the ~0.10–0.20 range, reflecting sizable deviations in reconstructed current height and shape (Table S4). For instance, in the representative 3 → 5 → 2 mV/s configuration, CV prediction yields Test_R^2^ values about ~0.89, substantially lower than those obtained for concentration and DCVA current. Unlike concentrations and DCVA, the experimental CV current integrates several scan-rate-dependent contributions that may not be directly correlated with the absorbance data. These contributions render the absorbance-to-current mapping effectively nonlinear and highly condition-dependent when only a single electrochemical trajectory is available for training.

**Figure 3:**
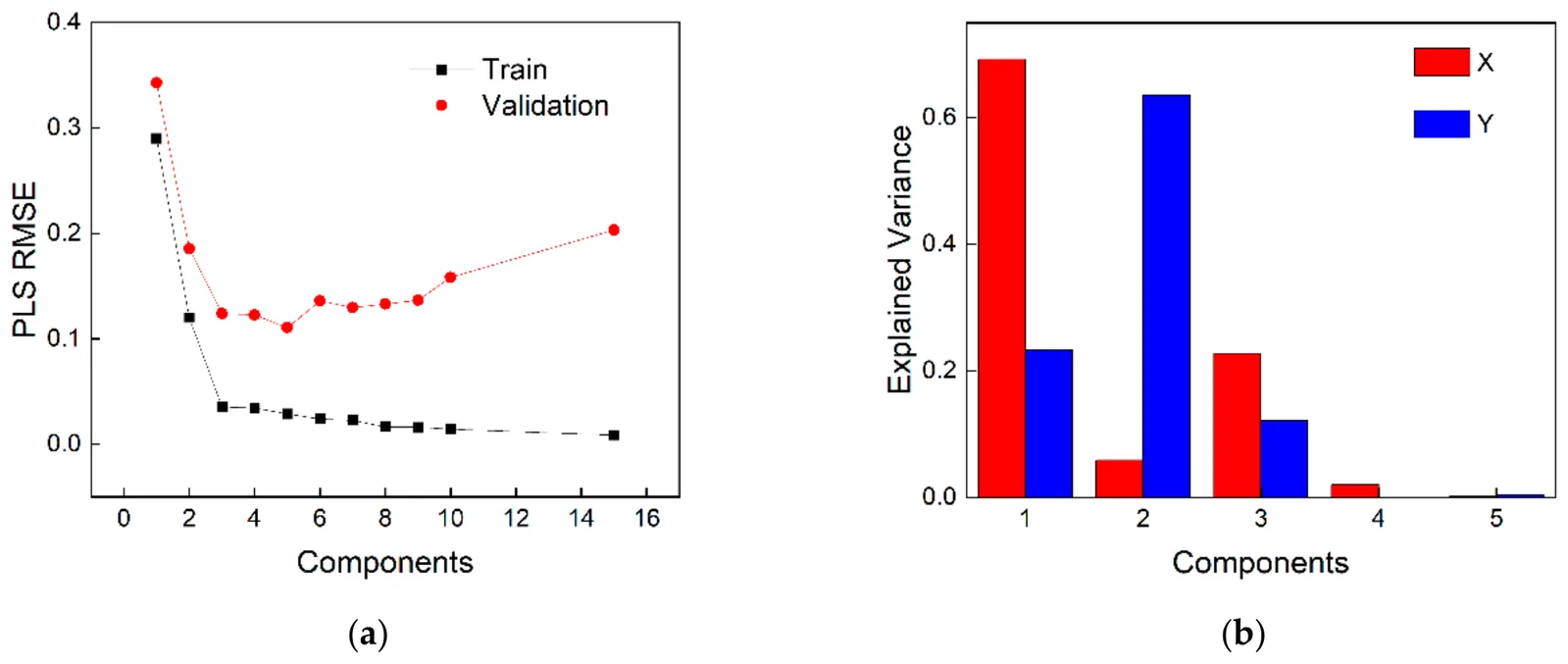
(**a**) PLS CV Single-SEC dataset training and validation RMSE as a function of components. (**b**) Explained variance for X and Y as a function of PLS components.

The behavior of the PLS model in this regime is most clearly elucidated by examining the nature of its components (latent variables), explained variance trends, and the structure of the X and Y loadings and scores. As illustrated in Figure 3a, the number of components increases from one upward, while validation RMSE decreases steeply over the first few components, for example, dropping from ~0.35 at one component to ~0.13 by three components in the representative 3 → 5 → 2 mV/s configuration, reflecting rapid capture of dominant, transferable spectroelectrochemical structure encoded in the absorbance data. This improvement saturates quickly after approximately three to five components, beyond which validation RMSE plateaus. Validation RMSE remains near ~0.11 between three and five components and increases marginally to ~0.12–0.13 at higher components. In practical terms, adding components beyond this range yields only marginal reductions in training error. While validation RMSE increases to ~0.15 at ten components, consistent with overfitting the CV current. Such behavior marks a transition from learning chemically meaningful variance that generalizes across scan rates to fitting residual structure that is specific to the training SEC dataset and does not transfer to unseen electrochemical conditions. This pattern is consistent with classical chemometric observations for systems in which predictors and responses are linked through significant nonlinearity, where linear prediction reaches a ceiling once physically transferable information has been exhausted [31].

Explained variance analysis reinforces this interpretation by revealing a pronounced difference between predictor and response spaces. Figure 3b highlights an asymmetry between how variance accumulates in the predictor (absorbance) and response (CV current) spaces, as the number of PLS components increases. Variance in the CV current space saturates substantially earlier than variance in absorbance space. The first two to three PLS components capture the dominant fraction of the explained CV variance, on the order of 80–90%. Beyond this point, additional components contribute only marginally to CV variance. In contrast, variance in the absorbance space continues to accumulate more significantly across higher components. While the first component already explains a large fraction of absorbance variance, the cumulative X variance rises from roughly ~70% to >80% by the fourth or fifth component. Importantly, this continued increase in absorbance variance does not translate into improved predictive CV performance for CV current. As such, validation RMSE remains effectively unchanged and plateaus around ~0.11 over this component range. This decoupling between X and Y variance growth provides a quantitative measure of the linear model’s limitation under single-SEC dataset training. Although the absorbance matrix is high-dimensional (while also highly collinear), not all spectral variance is electrochemically informative. In particular, scan-rate-dependent capacitive currents, baseline curvature due to background (solvent) decomposition, and hysteresis contribute strongly to the experimental CV signal but are not encoded in the absorbance data. Consequently, capturing additional spectral variance through higher PLS components does not translate into improved predictability of CV current within a linear modeling framework.

Interpretation of the X loadings provides further insight into the chemical content of the learned latent variables (Figure S4). The loadings correspond to mean-centered absorbance data, emphasizing relative spectral contrasts rather than absolute absorbance. Moreover, because PLS latent variables are optimized to maximize covariance with the response rather than mutual orthogonality, they should not be interpreted as pure species spectra in the same sense as components obtained from PCA or evolving factor analysis [33]. Instead, they represent chemically informed mixtures that emphasize spectral features most relevant to predicting the electrochemical response.

The first PLS component is dominated by features characteristic of the anthraquinone dianion, including a strong band centered near ~500 nm and a broad envelope extending from approximately 550 to 800 nm [34]. These features closely match the AQ^2−^ dianion (DAQ) spectrum identified previously via EFA and reflect the strong covariance between dianion population and CV current magnitude as the system enters the second reduction wave. In addition to the positive absorbance, this first component also exhibits a significant negative feature near 320–330 nm, which is consistent with the absorbance range of neutral AQ (NAQ). Arising from mean-centering, this negative feature is consistent with the depletion of NAQ with the growth of other reduced species along the voltammetric scan. This component alone accounts for a substantial fraction of the explained variance in the CV current and drives much of the initial decrease in validation RMSE.

The second component introduces spectral features associated with the radical anion, including a sharper band near ~400 nm, a secondary feature around ~540 nm, and a broad delocalized tail extending into the near-infrared region between ~650 and 850 nm [35,36]. These signatures align well with the established radical anion AQ^•−^ (RAQ) spectrum and capture the contribution of the first reduction step to the electrochemical response, particularly in the region where the two redox waves overlap [29]. Together, the first two components span the chemically dominant redox subspace of the system and encode the principal spectroscopic drivers of faradaic current. In contrast, higher-order components exhibit weaker, more fragmented spectral features with no clear correspondence to a single redox species. These components typically contain broad, low-amplitude contrasts distributed across multiple wavelength regions, reflecting mixed or residual contributions. The spectral features of neutral anthraquinone also appear diffusely embedded across these higher components rather than isolated in a dominant latent variable. This is consistent with both mean-centering preprocessing and the PLS deconvolution, which deprioritizes variance that does not covary strongly with the response [31].

As illustrated in Figure S5, the structure of the Y loadings reveals a hierarchical contribution to CV current prediction. The second PLS component carries the dominant positive loading in the CV space and accounts for the majority of the explained current variance, indicating that it captures the primary redox-driven contribution to current magnitude that transfers across scan rates. In contrast, the remaining components (1, 3, 4, and 5) exhibit negative (anti-correlation) Y loadings and substantially smaller magnitudes, reflecting anti-correlated and corrective contributions rather than independent current-driving processes. Among these, components 1, 3, and 4 have minor contributions, while the fifth component shows a modestly larger negative contribution, consistent with residual structure.

**Figure 4:**
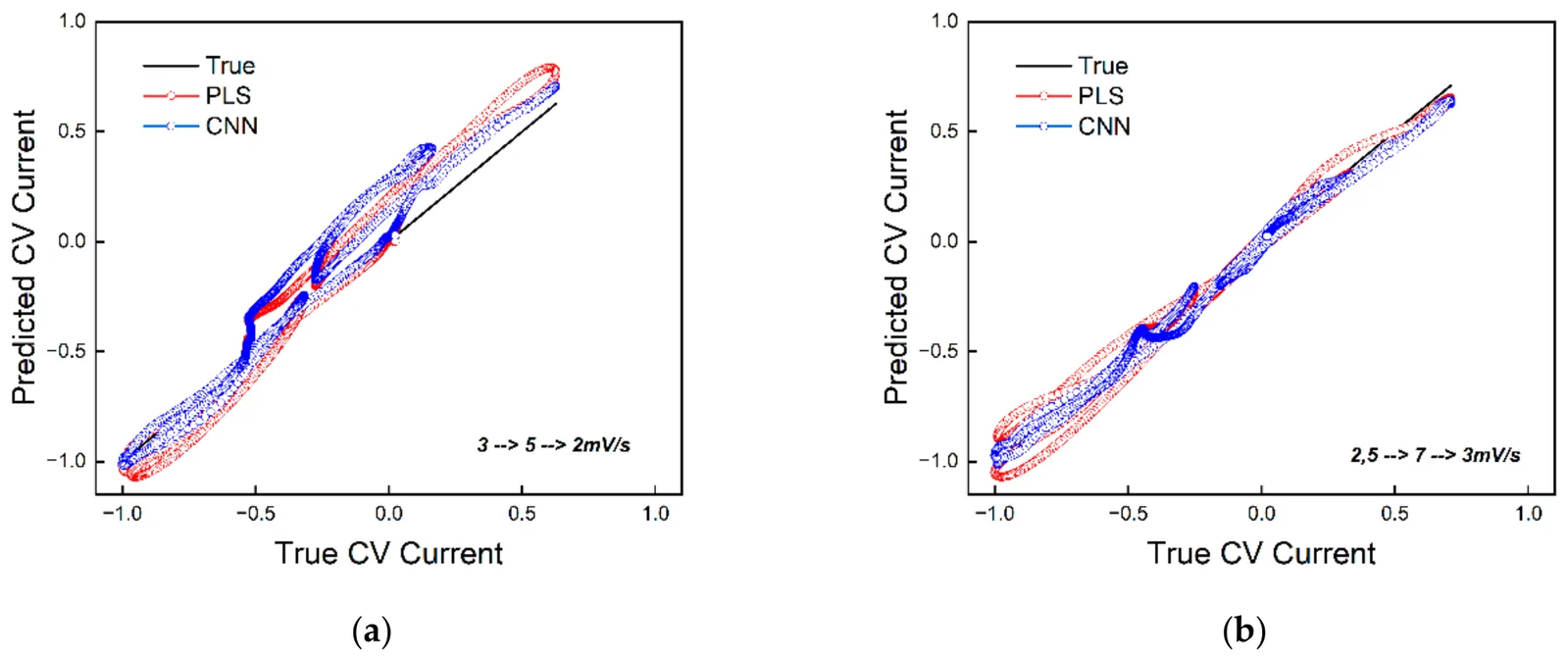
(**a**) PLS and CNN CV parity plot for single-SEC dataset training of the 3 → 5 → 2 mV/s configuration, (**b**) PLS and CNN CV parity plot for of pair-SEC training of the 2;5 → 7 → 3 mV/s configuration.

These trends are also reflected directly in the qualitative output of the PLS model. Reconstructed CV current reproduces peak positions and approximate peak height, yet consistently under-captures peak sharpness, baseline curvature, and scan-rate-dependent distortions. When applied to the unseen test SEC dataset, these deficiencies become more pronounced. As such, parity scatter broadens, and reconstruction errors accumulate in regions dominated by capacitive and background contributions, underscoring the limited extrapolation capacity of single-SEC dataset linear models (Figure 4a). The first few latent variables capture chemically meaningful, transferable redox information that generalizes across scan rates, while additional components increasingly model scan-rate-specific distortions that cannot be linearly inferred from absorbance alone.

### 2.3. CNN Performance in the Single-SEC Regime

For concentrations (Table S1), the CNN achieves the same near-quantitative ceiling as PLS, with Test_R^2^ values consistently ~0.99 across single-SEC configurations and corresponding Test_RMSE values remaining very low (typically on the order of ~0.01). As shown in the parity plot (Figure S3), CNN prediction of concentration profiles is indistinguishable from PLS within experimental noise. For DCVA current prediction (Figure 2b), CNN performance under single-SEC dataset training is in many configurations modestly higher than PLS. As shown in Table S2, typical Test_R^2^ values lie in the 0.90–0.98 range, with Test_RMSE values reduced slightly relative to PLS. These improvements are consistent with the CNN’s ability to exploit local potential-neighborhood structure, which is particularly relevant for derivative-based quantities such as DCVA. Nevertheless, the gains remain bounded in the single-SEC regime. Although locality helps stabilize derivative features, a single SEC dataset still only partially specifies how DCVA peak shape, spacing, and amplitude deform with scan rate.

The CNN’s most meaningful advantage under single-SEC dataset training appears in CV current prediction. CNN CV performance was first evaluated through model optimization and diagnostic analysis. A hyperparameter sweep over convolutional width (number of filters), kernel size, dense-layer dimensionality, and regularization strength reveals an optimum rather than a monotonic improvement with increasing model complexity (Figure S6). Using the 3 → 5 → 2 mV/s split, the lowest validation RMSE is obtained for configuration 37 (128 filters, kernel size 9, 64 dense units, no dropout, no L2 regularization), with validation RMSE reduced to ~0.10 compared to ~0.12–0.14 for smaller or strongly regularized models (Table S3). Larger architectures (e.g., 256 filters or wider dense layers) do not yield additional improvements and instead slightly increase validation error. Under the present experimentally scarce setting, the validation space itself is necessarily limited to a single SEC dataset, and therefore hyperparameter selection may partially bias absolute performance estimates toward the chosen validation scan rate. Consequently, the optimized CNN configuration should not be interpreted as a uniquely generalizable architecture, but rather as evidence that a finite nonlinear capacity window exists in which localized spectral–electrochemical correlations can be learned without substantial degradation of transferability. This behavior contrasts with PLS, where saturation arises because additional latent variables primarily increase sensitivity to non-transferable variance, whereas CNN architectural tuning defines a bounded nonlinear representation capacity capable of capturing localized scan-rate-conditioned structure without destabilizing extrapolation.

The loss versus epoch curve (Figure S7) for the selected CNN model shows smooth and rapid convergence, typically stabilizing within ~20–30 epochs, with training and validation losses tracking closely. Test parity plots (Figure 4a) show slightly tighter clustering around the parity line than observed for PLS, particularly at intermediate and high absolute currents, where the linear model exhibits systematic underestimation. SEC-level reconstructions reproduce peak positions and overall current scale while recovering more of the scan-rate-dependent curvature and anodic-cathodic asymmetry that PLS tends to attenuate. Although errors persist at extreme potentials and in regions where non-faradaic contributions dominate, the CNN shifts prediction performance beyond the apparent linear saturation limit of PLS by learning localized, scan-rate-conditioned correlations between spectral evolution, potential progression, and the overall experimental current waveform, rather than by directly spectroscopically encoding double-layer charging itself.

Across several single-SEC dataset training combinations, CNN CV Test_R^2^ values exceed those obtained with PLS for the same train-validation-test splits (Tables S4 and S5). CNN Test_R^2^ values fall to about ~0.80 on average, compared to ~0.77 for PLS, with slight corresponding reductions in Test_RMSE. These gains indicate that the CNN is able to recover additional transferable structure even when trained on a single electrochemical trajectory.

Viewed collectively, the single-SEC dataset results reveal a consistent progression in model behavior as the electrochemical target increases in complexity. For concentration prediction, both PLS and CNN converge to the same near-quantitative ceiling, with Test_R^2^ values ≈ 0.99 and minimal Test_RMSE across all single-SEC dataset configurations. This convergence confirms that, when Beer–Lambert linearity dominates and scan-rate dependence is weak, nonlinear model capacity provides no intrinsic advantage. In this regime, a single SEC dataset already spans a sufficiently complete absorbance-concentration subspace, and both models operate close to their best performance. As the target shifts to DCVA current, performance declines to an average Test_R^2^ value ~0.93, reflecting the derivative nature of DCVA and its sensitivity to scan-rate-dependent peak deformation. Here, the CNN exhibits modest but consistent gains relative to PLS by exploiting local correlations along the potential axis that are emphasized by differentiation. Nevertheless, both models remain fundamentally constrained by the same limitation, where a single electrochemical trajectory does not fully specify how derivative features evolve when scan rate changes. As a result, CNN locality improves stability but does not fundamentally alter the performance ceiling in the single-SEC regime.

The clearest divergence between linear and nonlinear modeling appears for experimental CV current. In this case, PLS performance saturates early, with Test_R^2^ typically dispersed in the ~0.65–0.90 range and validation RMSE plateauing once the dominant redox-related variance is captured. This saturation reflects the inability of a global linear latent space to encode scan-rate-dependent capacitive currents, baseline curvature, and hysteresis that are weakly represented in the absorbance data [37,38]. The CNN, by contrast, seems to be more consistent in CV learnability, achieving a higher average Test_R^2^ value of 0.80 (as compared to 0.77 for PLS) under single-SEC dataset training. These gains arise not from increased data volume, but from the CNN’s ability to learn localized nonlinear mappings within potential windows that couple spectral evolution and electrochemical response more flexibly than a global projection. These single-SEC dataset results show that concentration prediction is already information-complete, that DCVA lies near the limit of what can be learned from one trajectory, and that CV prediction remains fundamentally underdetermined without explicit exposure to scan-rate variation. The key question, therefore, is not whether additional model flexibility alone can overcome these constraints, but whether introducing a second, distinct electrochemical trajectory provides sufficient structured diversity to resolve scan-rate-dependent distortions and allow each model to approach its practical performance ceiling.

### 2.4. Paired-SEC Mapping

As discussed above, single-SEC dataset training constrains learning to one electrochemical trajectory and therefore cannot fully describe how spectroscopic-electrochemical relationships evolve with scan rate. Rather than expanding the dataset in a conventional sense, paired-SEC dataset training introduces the smallest meaningful extension beyond the single-SEC dataset limit. As such, two SEC datasets acquired at distinct scan rates are used for training, while validation and testing are performed on fully unseen SEC datasets. This design preserves the low-rank character of the problem while supplying a second electrochemical condition, enabling the models to distinguish scan-rate-invariant spectroscopic structure from scan-rate-dependent current contributions.

#### 2.4.1. PLS with Paired Training SEC Datasets

For concentrations, PLS remains near-quantitative regardless of training size, with Test_R^2^ values consistently near ~0.99 across all single- and paired-SEC dataset configurations. Correspondingly, Test_RMSE values remain very small and largely invariant, typically on the order of a few percent of the total concentration range. Paired-SEC datasets training does not reduce Test_RMSE appreciably relative to single-SEC dataset training, confirming again that concentration prediction is already saturated under minimal training diversity. For DCVA, paired-SEC datasets training produces a consistent and systematic elevation in performance relative to the single-SEC regime. Whereas single-SEC dataset PLS DCVA Test_R^2^ values typically fall in the ~0.87–0.97 range, paired-SEC datasets training shifts typical performance into the ~0.90–0.98 range, with the highest gains observed when the two training SEC datasets are meaningfully separated in scan rate (Table S2).

**Figure 5:**
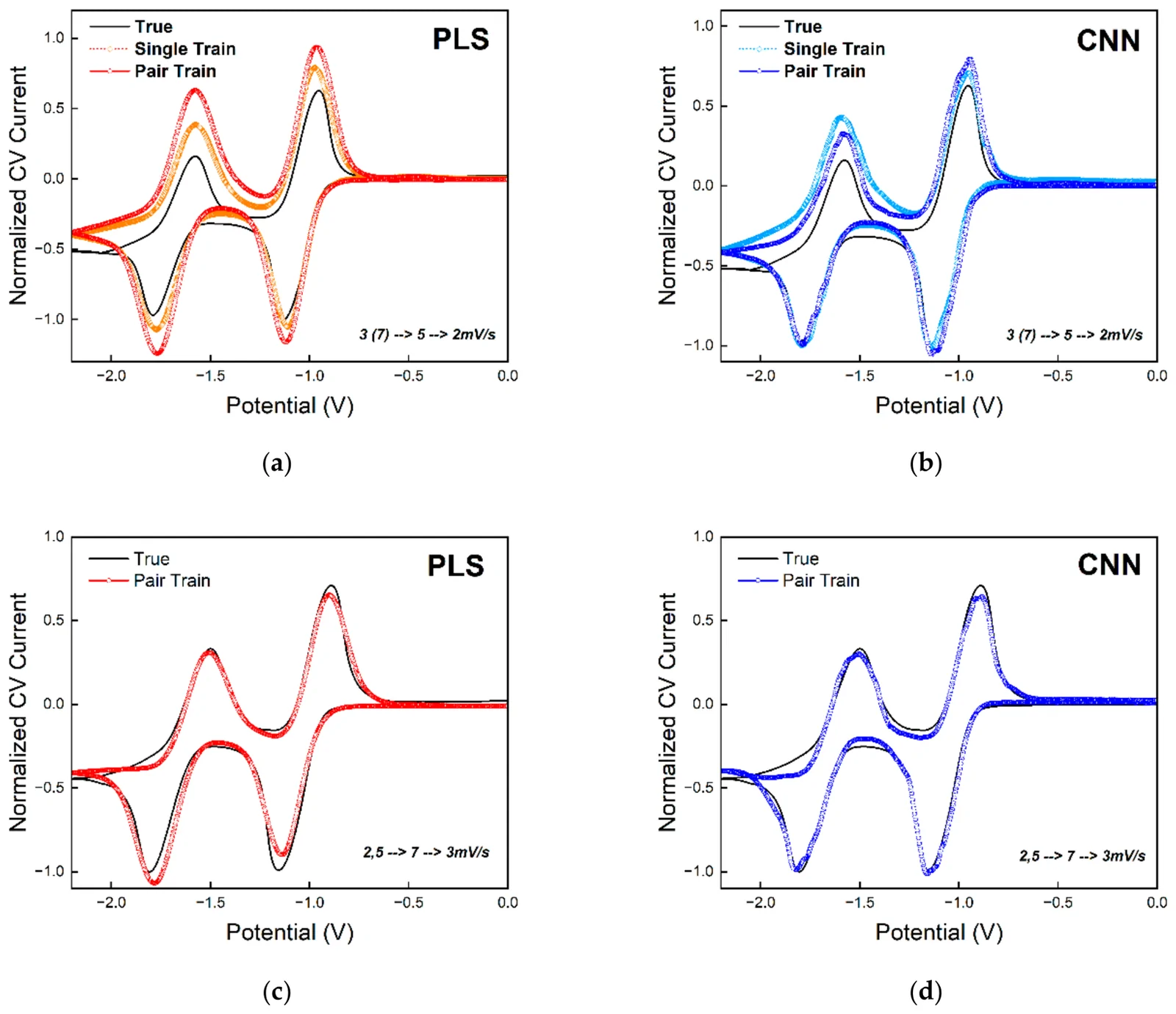
(**a**) PLS and (**b**) CNN CV predictions from single and pair SEC datasets training for the 3 (7) → 5 → 2 mV/s. (**c**) PLS and (**d**) CNN CV predictions from pair SEC datasets training for the 2;5 → 7 → 3 mV/s.

For CV current, paired-SEC dataset training produces the most dramatic change in PLS performance. Upon introducing a second training SEC dataset, combinations that span large scan-rate separation yield Test_R^2^ values approaching ~0.98. Average CV Test_RMSE values decreased from ~0.063 in the single-SEC regime to ~0.051. Figure 5c shows a marked reduction in systematic reconstruction error across the voltammogram, for the 2;5 → 7 → 3 mV/s configuration. In the pair-mapping plots, this transition manifests as a clear shift from fragmented regions of alternating high and low performance under single-SEC dataset training to broad plateaus of good performance when the training SEC datasets bracket the test condition. Mechanistically, the second training SEC dataset expands the latent variable space sufficiently for PLS to encode scan-rate-dependent changes in peak shape, peak width, and baseline curvature, features that appear nonlinear when viewed from any single trajectory but become substantially linear when anchored at two distinct scan rates.

#### 2.4.2. CNN with Paired Training SEC Datasets

Under paired-SEC datasets training, the CNN exhibits a clear and consistent improvement across all targets, with the most pronounced gains observed for experimental CV current prediction. As in the PLS case, concentration prediction remains uniformly high and essentially invariant with respect to training diversity, with Test_R^2^ values remaining near unity across all single- and paired-SEC dataset configurations and corresponding Test_RMSE values remaining very small. For DCVA, however, CNN performance improves more than PLS upon introduction of a second training SEC dataset. Whereas single-SEC dataset CNN DCVA Test_R^2^ values typically lie in the ~0.90–0.98 range, paired-SEC dataset training raises these values to ~0.97–0.99 for most scan-rate combinations. Additionally, average Test_RMSE decreases from ~0.07 in single-SEC dataset training to about ~0.05 in the pair training setting.

The largest distinction between CNN and PLS emerges in paired-SEC datasets CV prediction. Using the optimized CNN configuration (configuration 37), paired-SEC datasets training yields Test_R^2^ values consistently in the ~0.80–0.93 range across a wide span of scan-rate combinations, frequently exceeding the PLS results obtained under selected pairings. Average CNN CV Test_R^2^ value of ~0.85 is accompanied by an average Test_RMSE value that decreases to ~0.12, compared to ~0.15 under single-SEC dataset training. More importantly, this high performance is achieved with substantially reduced sensitivity to the exact choice of training SEC datasets. Whereas PLS CV performance is maximized primarily when training SEC datasets bracket the test scan rate, CNN CV performance remains high even for less extreme pairings. For example, Test_R^2^ remains above ~0.85 for training pairs separated by 1–3 mV/s, while PLS values fall below this threshold. This contrast is also illustrated directly by the behavior of the 3 → 5 → 2 mV/s split (Figure 5a,b). In the single-SEC dataset regime, PLS exhibits a marked drop in CV prediction performance when the 7 mV/s SEC is added as a second training scan, reflecting sensitivity to the introduction of highly non-transferable scan-rate-dependent structure. In contrast, the CNN shows the opposite response: inclusion of the 7 mV/s SEC dataset improves performance, tightening parity and reducing Test_RMSE, consistent with the network using the additional scan-rate anchor to learn scan-rate-conditioned local nonlinear mappings rather than destabilizing a global representation. This divergent response highlights the CNN’s ability to distribute complexity locally across potential regions, enabling robust interpolation once minimal experimental diversity is introduced.

**Figure 6:**
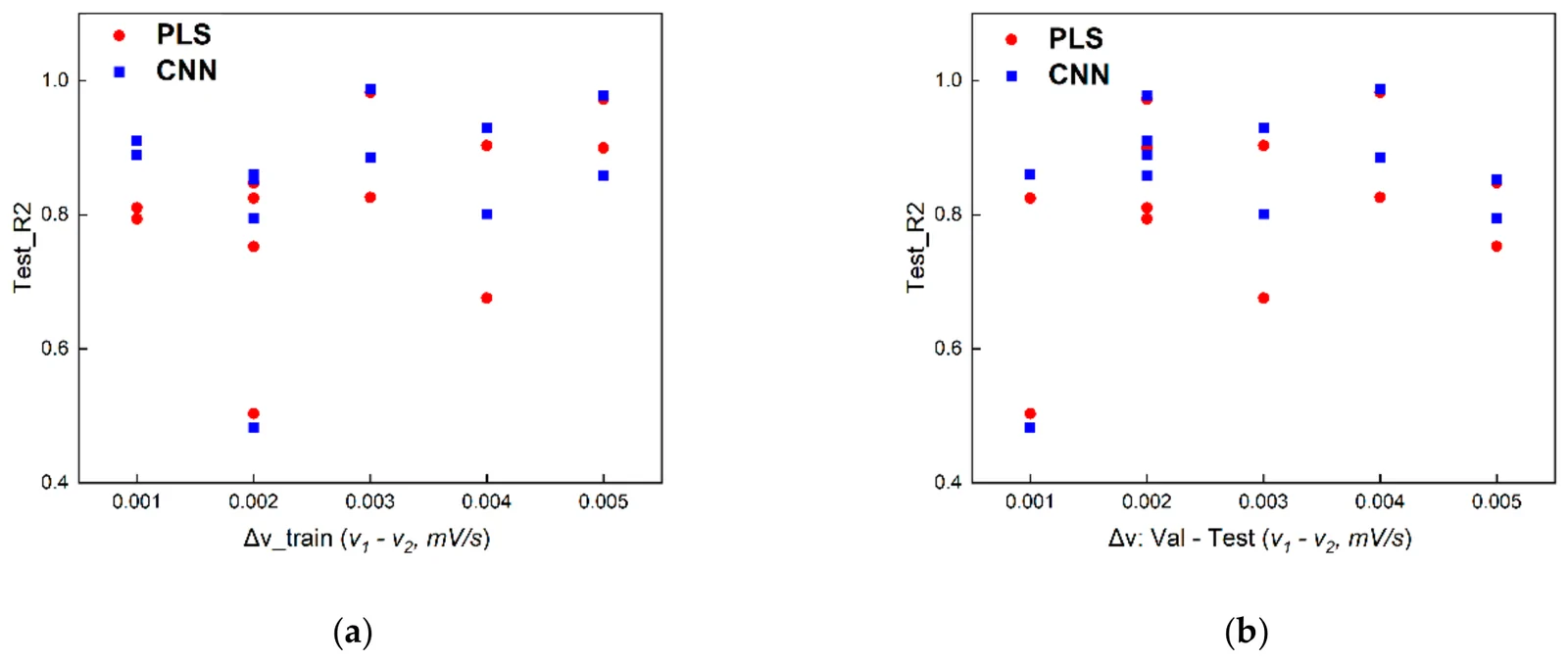
PLS and CNN mapping performance in terms of Test_R2 from pair-SEC dataset training as a function of (**a**) training scan rate difference and (**b**) validation and test scan rate difference.

This trend is further supported by the quantitative relationship between CNN test performance and the scan-rate separation Δv between training and validation-test SEC datasets (Figure 6a,b). For instance, Test R^2^ remains relatively stable within the ~0.80–0.93 range over a broad Δv window, while Test RMSE varies smoothly by only a few hundredths rather than exhibiting abrupt increases. In contrast, PLS performance is more dispersed across comparable Δv windows in the paired-SEC configurations. Parity plots (Figure 4b) further illustrate this behavior, showing tighter clustering around the parity line with slopes approaching unity and minimal systematic bias for representative CNN predictions. For example, the average CNN Test RMSE decreases from approximately 0.15 ± 0.05 under single-SEC training to 0.12 ± 0.05 under paired-SEC training (Table 1). Reconstructed paired-SEC CNN CV traces further demonstrate improved recovery of peak position, peak asymmetry, baseline curvature, and forward–reverse hysteresis relative to both single-SEC CNN and paired-SEC PLS predictions, particularly in regions where capacitive and scan-rate-dependent distortions contribute strongly to the experimental current response. Although the average performance differences between PLS and CNN are comparable to the permutation-to-permutation variability, the same qualitative trends remain consistently observable across the complete set of train/validation/test permutations reported in the Supplementary Materials (Tables S4 and S5), supporting the proposed interpretation rather than a definitive ranking of the two models based solely on average performance metrics. Table 1 summarizes the mean and standard deviation of the obtained test metrics for both models under single- and paired-SEC training.

Overall, the paired-SEC results resolve the apparent limitations observed under single-SEC training and illustrate the respective behavior of linear and nonlinear learning. The dominant constraint revealed by single-SEC training is not insufficient model capacity, but incomplete sampling of electrochemical condition space. Once two distinct electrochemical trajectories are provided, both PLS and CNN models approach a similar practical performance ceiling for CV prediction, indicating that the spectroscopic inputs already encode most of the transferable information required to reconstruct the electrochemical response when appropriately conditioned. Within the scope of the present comparison, the primary distinction therefore lies in generalization robustness rather than absolute prediction accuracy [27]. PLS achieves near-quantitative performance when training SEC datasets are deliberately selected to maximize electrochemical contrast but remains more sensitive to pairing choices due to its reliance on a limited set of global latent variables. In contrast, the CNN distributes complexity across localized nonlinear filters, reducing dependence on precise experimental pairing and yielding smoother, more uniform generalization across scan-rate space. It is worth noting, however, that the present study was intentionally designed to compare a classical linear chemometric framework with a representative nonlinear deep-learning architecture, rather than to exhaustively benchmark all possible intermediate nonlinear models. Consequently, the observed CNN advantages should be interpreted as evidence that localized nonlinear representations can improve robustness under SEC scarcity, within the experimental conditions investigated here, rather than as definitive proof that convolutional architectures are uniquely optimal relative to other nonlinear approaches such as kernel methods or shallow neural networks.

## 3. Materials and Methods

### 3.1. Experimental

Anthraquinone (AQ, 9,10-anthraquinone, 97%), tetrabutylammonium hexafluorophosphate (TBAPF_6_, ≥99% electrochemical grade), and anhydrous acetonitrile (ACN, 99.8%) were purchased from Sigma-Aldrich (St. Louis, MO, USA) and used as received. All spectroelectrochemical solutions (3 mM AQ and 0.1 M TBAPF_6_) were prepared and handled inside an argon-filled glovebox with oxygen and water levels maintained below 1 ppm. SEC measurements were performed using a low-volume thin-layer (0.5 mm light pathlength) quartz cell (BASi Instruments, West Lafayette, IN, USA). A platinum mesh served as the working electrode, with a platinum wire counter electrode and a silver wire pseudo-reference electrode. The SEC cell was fully assembled and sealed inside the glovebox prior to experiments to prevent air exposure. UV-visible-NIR spectra were recorded using a SPELEC instrument (Metrohm Dropsens, Asturias, Spain), controlled through DropView SPELEC software (https://metrohm-dropsens.com/products/software/dropview-spelec-software-2/ (accessed on 7 August 2024)). SEC optical acquisition parameters included an integration time of 20 ms and a number of scans to average 25, while electrochemical conditions contained the forward and backward CV potential range (0 to −2.2 V) and recorded scan rate (2, 3, 5 and 7 mV/s) for each experiment. All SEC experiments were conducted under identical solution composition and geometry, with scan rate as the primary controlled experimental variable. Change in absorbance as a function of applied potential at selected wavelengths, across the four studied scan rates, is shown in Figure S8. More data plots of experimental SEC could be found in the Supporting Information of previous work [29].

### 3.2. SEC Experimental Design

Previously extracted concentration and DCVA profiles via physics-based processing of SEC data are used as reference targets in this work for machine-learning models [29]. A single SEC experiment consists of full spectral absorbance and cyclic voltammetric current, including forward and reverse scans (0 to −2.2 V). At each potential, a wavelength-resolved absorbance spectrum spanning the 300–850 nm range is recorded simultaneously with the electrochemical current. Each SEC unit therefore forms a multi-dimensional unit linking potential, spectral evolution, and current. Four independent SEC datasets acquired at distinct scan rates (2, 3, 5, and 7 mV s^−1^) are employed in this study. One important consideration for the present AQ system, extensively discussed in the previous mechanistic study [29], is that the experimentally accessible scan-rate window is intrinsically constrained by competing physicochemical effects. At very slow scan rates (~1 mV s^−1^), significant anthraquinone radical-anion dimerization and other homogeneous chemical reactions begin to perturb the reversible SEC response, whereas higher scan rates progressively introduce uncompensated resistance, mass-transport limitations associated with the SEC cell geometry and electrode surface area, and increased capacitive distortions. The selected 2–7 mV s^−1^ range therefore represents the most experimentally reliable window for preserving chemically reversible behavior while introducing controlled scan-rate-dependent kinetic variability appropriate for the present learning analysis. Within this range, the study specifically examines whether limited but strategically introduced experimental diversity is sufficient to support transferable learning across independent SEC datasets. Throughout this work, experimental scarcity refers to the limited availability of independent SEC experiments rather than the number of measurements contained within each experiment. Each SEC dataset comprises a high-dimensional absorbance matrix sampled over hundreds of potentials and wavelengths, providing abundant within-experiment information but representing only a single set of electrochemical conditions. Consequently, the primary limitation is not matrix dimensionality, but the limited diversity of independent experimental conditions, particularly scan-rate-dependent kinetics, available for model training and validation. In the present study, the four experimentally acquired SEC datasets constitute the complete experimental space. The central objective is therefore to determine how much electrochemical information can be learned from a minimal number of independent SEC experiments and how the diversity of electrochemical conditions influences model generalization. Thus, the present study specifically investigates whether limited but strategically introduced experimental diversity is sufficient to support transferable learning.

### 3.3. Computational

All data-driven analysis and evaluation were performed using Python-based open-source tools executed in a controlled local environment (Python 3.9, Anaconda 24.5.0). Numerical operations and data handling were performed using NumPy and Pandas, while visualization was performed using Python Matplotlib and Origin software (2024b). Linear modeling employed scikit-learn implementations of partial least squares regression, and deep learning models were constructed using TensorFlow/Keras. Two regression models, partial least squares regression (PLS) and convolutional neural networks (CNN), are compared under identical preprocessing, splitting, and evaluation protocols. More details on data dimensionality, preprocessing, and computational workflow are provided in the Section 2 and in the Supplementary Materials.

## 4. Conclusions

This work demonstrates that direct learning and calibration of electrochemical targets from spectroelectrochemical absorbance data is achievable even under experimentally realistic data scarcity within a chemically reversible and spectroscopically well-behaved SEC system. Using both a linear regressor and a nonlinear deep-learning framework, a consistent hierarchy emerges across outputs of increasing physical complexity, providing insight into how target structure, experimental diversity, and model choice collectively govern learnability in low-data spectroelectrochemical analysis. Species concentrations are predicted near-quantitatively even with a single SEC dataset, establishing a baseline for SEC learnability and reflecting the strong linear structure imposed by Beer–Lambert behavior and the underlying factorial decomposition. In contrast, DCVA current introduces greater prediction complexity because numerical differentiation amplifies experimental noise and potential-dependent variations, leading to moderate performance loss under single-SEC training. Experimental CV current represents the most stringent test, as it integrates faradaic processes with scan-rate-dependent capacitive, background, and hysteretic contributions that are weakly encoded in the optical signal. Model comparisons clarify both the source of these limitations and how they can be overcome. Under single-SEC dataset training, PLS provides a transparent linear baseline and reveals early saturation for CV prediction. Once a small number of latent variables capture the dominant redox-related covariance, additional components primarily fit scan-rate-specific residual structure that does not readily transfer across SEC datasets. CNNs partially extend this linear performance ceiling by learning localized nonlinear relationships within potential windows, improving CV reconstruction relative to PLS when only one SEC dataset is available. Within the present AQ SEC system, introducing just two SEC datasets acquired at distinct scan rates enables both PLS and CNN regressors to generalize strongly to an unseen third SEC dataset, with CV Test_R^2^ values approaching ~0.97–0.98 in the best-performing configurations. Under this paired-SEC training regime, much of the apparent nonlinearity observed under single-SEC training is better understood as a consequence of limited experimental sampling, and the spectroscopic–electrochemical mapping becomes substantially more linear once multiple electrochemical trajectories anchor the latent space. Both models consequently approach a similar practical performance ceiling, while the CNN exhibits greater robustness to the choice of training SEC pair and more consistent generalization across scan-rate combinations.

From an application perspective, learning directly from experimental SEC data is highly relevant to calibration, sensing, and process-monitoring problems, where robust electrochemical prediction is required under changing operating conditions using rich optical fingerprints. In a broader analytical scenario, such mapping would enable indirect electrochemical estimation from optical measurements, reconstruction or denoising of voltammetric responses, and recovery of chemically meaningful current components that may otherwise be obscured by capacitive or kinetic distortions in measured voltammograms. From a modeling perspective, which is the main scope of this work, SEC data processing provides a uniquely constrained testbed for a broader data-science question about when coupled spectroscopic-electrochemical datasets can be treated as effectively linear, and where nonlinearity becomes unavoidable. The present findings show that (non)linearity is inherently target-dependent and becomes operationally significant only when scan-rate space is sufficiently sampled. As a consequence, the most efficient experimental strategy is not to collect many redundant SEC datasets, but to acquire a small number that span selected kinetic conditions. Within this regression framework, PLS serves as an interpretable baseline for quantifying the linearly recoverable fraction of the signal, while CNN provides a robust extension when accurate CV current reconstruction across conditions is desired. The choice between classical chemometric approaches and modern deep learning models is therefore governed not by SEC data volume alone, but by target complexity, the degree of condition (scan-rate) dependence, and experimental feasibility.

## Figures and Tables

**Table 1:** Mean and standard deviation test metrics of PLS and CNN across all targets.

Targets		PLS		CNN	
		RMSE	R^2^	RMSE	R^2^
Concentration	Single	0.017 ± 0.0047	0.999 ± 0.0006	0.030 ± 0.0097	0.996 ± 0.0026
DCVA	Single	0.080 ± 0.0247	0.927 ± 0.0518	0.072 ± 0.0238	0.942 ± 0.0363
	Pair	0.074 ± 0.0212	0.938 ± 0.0386	0.049 ± 0.0150	0.973 ± 0.0169
CV	Single	0.153 ± 0.0625	0.77 ± 0.21	0.147 ± 0.0525	0.80 ± 0.14
	Pair	0.139 ± 0.0509	0.82 ± 0.13	0.123 ± 0.0519	0.85 ± 0.13

## Data Availability

The original contributions presented in this study are included in the article/Supplementary Materials. Further inquiries can be directed to the author.

## Supplementary Materials

The following supporting information can be downloaded at: https://www.mdpi.com/article/10.3390/molecules31162836/s1, SEC Data Preprocessing, Figure S1: Preprocessing screening for PLS on both X and Y data, Figure S2: Experimental SEC-CV currents, recorded at different scan rates, Figure S3: Parity plot of concentration (C1) values, comparing true values with single-SEC trained PLS and CNN models prediction, Figure S4: X loading of the first and second PLS component, Figure S5: Y loading as a function of PLS components, Figure S6: CNN hyperparameter sweep optimization based on validation RMSE, Figure S7: CNN training and validation Losses as function of epochs, Figure S8: Change of absorbance at selected wavelengths as a function of applied potential across the four studied scan rates, Table S1: Single-SEC performance for concentration, Table S2: Single and pair SEC mapping performance for DCVA current, Table S3: CNN hyperparameters sweep optimization and configurations, Table S4: PLS single and pair SEC mapping performance for CV current, Table S5: CNN Single and pair SEC mapping performance for CV current.
