# Supplementary material for: Linear and Nonlinear Learning from Spectroelectrochemical Data: Interrogation of PLS and CNN Behavior Under Experimental Scarcity
Source: Molecules. 2026 Aug 14;31(16):2836. doi: 10.3390/molecules31162836 (PMC13515887; doi:10.3390/molecules31162836)
Supplement: Supplementary file 1 [file molecules-31-02836-s001.zip › molecules-4447445-supplementary.pdf]

Supplementary Information

Linear and Nonlinear Learning from Spectroelectrochemical Data: Interrogation of PLS and CNN Behavior under Experimental Scarcity

Abderrahman Atifi*

Energy and Environment Science & Technology, Idaho National Laboratory, Idaho Falls, ID 83415, USA

Corresponding author E-mail: abderrahman.atifi@inl.gov

Table of Contents:

- *SEC Data Preprocessing*
- *Figure S1: Preprocessing screening for PLS on both X and Y data*
- *Figure S2: Experimental SEC-CV currents, recorded at different scan rates*
- *Figure S3: Parity plot of concentration (C1) values, comparing true values with single-SEC trained PLS and CNN model predictions.*
- *Figure S4: X loading of the first and second PLS components*
- *Figure S5: Y loading as a function of PLS components*
- *Figure S6: CNN hyperparameter sweep optimization based on validation RMSE*
- *Figure S7: CNN training and validation losses as a function of epochs.*
- *Figure S8: Change of absorbance at selected wavelengths as a function of applied potential across the four studied scan rates*
- *Table S1: Single-SEC performance for concentration*
- *Table S2: Single and pair SEC mapping performance for DCVA current*
- *Table S3: CNN hyperparameters sweep optimization and configurations*
- *Table S4: PLS single and pair SEC mapping performance for CV current*
- *Table S5: CNN Single and pair SEC mapping performance for CV current*

SEC Data Preprocessing

All targets were normalized to comparable ranges (concentrations to [0,1]; DCVA and CV currents to [-1,1]) to ensure that model optimization and performance metrics were not dominated by differences in absolute signal magnitudes. Scan rate v was treated as an explicit experimental covariate. Each supervised sample inherited the scan rate of its parent SEC, and the transformed variable s(v)=log(v) was appended as an auxiliary input to both PLS and CNN models, enabling scan-rate-conditioned prediction while preserving strict SEC-level generalization. Intentionally in the present study, normalizing the CV current suppresses the trivial amplitude scaling associated with faradaic current–scan-rate relationship. By removing dominant magnitude effects, current normalization allows generalization performance to be evaluated primarily in terms of waveform, peak shape, baseline curvature, and scan-rate-dependent distortions, rather than absolute current calibration. All data splitting is performed strictly at the SEC level to prevent information leakage. Entire SECs are assigned to training, validation, or test sets. Two learning regimes are considered: a single-SEC regime, in which one SEC is used for training and performance is evaluated on unseen SECs, and a paired-SEC regime, in which two SECs are used for training while validation and testing are performed on independent scans.

Several preprocessing methods were initially evaluated for both the predictor matrix (X) and target vector (Y), including no preprocessing, autoscaling (standardization), global maximum normalization (across SEC datasets), min–max scaling, mean-centering, standard normal variate (SNV), and combinations thereof (Figure S1). Screening was performed using a representative SEC split (training, validation, and test scan rates of 2, 5, and 3 mV s^{-1}, respectively), in which the test scan rate lies between the training and validation scan rates. For each preprocessing strategy, model performance was assessed using the validation RMSE. Across the evaluated methods, most preprocessing strategies produced very similar validation performance, with validation RMSE differing by approximately 0.01 for this representative split. This limited sensitivity is largely attributed to the fact that the experimental absorbance spectra had already undergone baseline correction during SEC data processing, while concentration and current targets were normalized prior to model development. Consequently, no additional smoothing, Savitzky–Golay derivatives, wavelength selection, or baseline correction were applied during model development. In contrast, SNV preprocessing of X produced a modest increase in validation RMSE, consistent with the removal of physically meaningful absorbance intensity variations that contribute to prediction. So, predictors were scaled by mean-centering and standardization according to $X_{sc}=(X-\mu_X)/\sigma_X$, and targets as $y_{sc}=(y-\mu_y)/\sigma_y$. All preprocessing parameters are fitted exclusively on training data and applied unchanged to validation and test sets.

CNN models were trained using the same SEC-level train/validation/test partitions as the PLS models. The network receives the SEC absorbance matrix as the primary input, while the scan rate, expressed as $\log_{10}(v)$, is included as an auxiliary experimental feature. No pooling layer was used in order to preserve potential-localized information across the voltammetric scan. The optimized CNN architecture consists of a one-dimensional convolutional layer with 128 filters and kernel size = 9, followed by a ReLU activation, feature flattening, concatenation with the scan-rate feature, a fully connected dense layer (64 neurons, ReLU), and a single linear

output neuron that predicts the electrochemical target at the corresponding potential. Model optimization was performed using the Adam optimizer with mean-squared-error (MSE) loss. Early stopping based on validation loss was applied to reduce overfitting, and the final model was selected using the minimum validation RMSE. Hyperparameters were optimized through a predefined grid search including:

- Convolutional filters: 64, 128, 256;
- Kernel size: 7, 9;
- Dense-layer width: 64, 128, 256;
- Dropout: 0, 0.2;
- L2 regularization: 0, 10^{-4}.

The optimal configuration corresponded to 128 filters, kernel size = 9, dense layer = 64 neurons, no dropout, and no L2 regularization.

CNN architecture:

Input SEC
|
▼
Conv1D (128 filters, k = 9)
|
ReLU activation
|
Feature Flatten
|
Concatenate scan-rate feature
s(v) = log10(v)
|
Dense Layer (64 neurons)
ReLU activation
|
▼
Linear Output (Predicted Target)

Training:
Adam optimizer • MSE loss • Early stopping
Model selection: Minimum validation RMSE

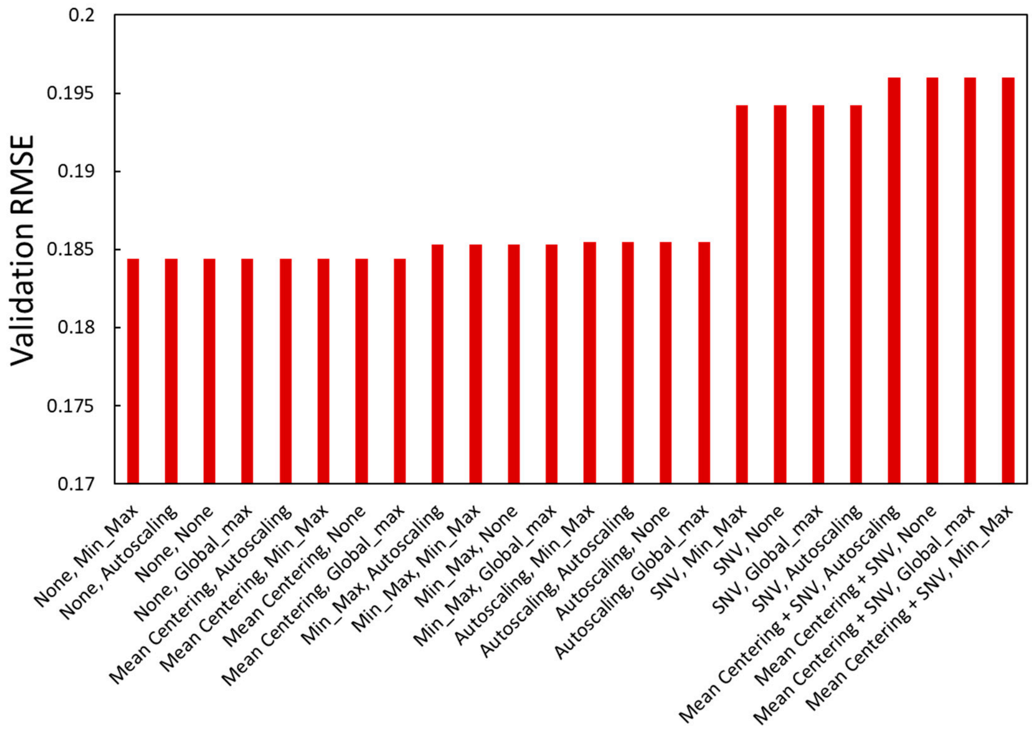

Figure S1: Preprocessing screening on both X (absorbance) and Y (CV) based on obtained validation RMSE for PLS single-SEC training, using 2, 5 and 3mV/s for training, validation and test split.

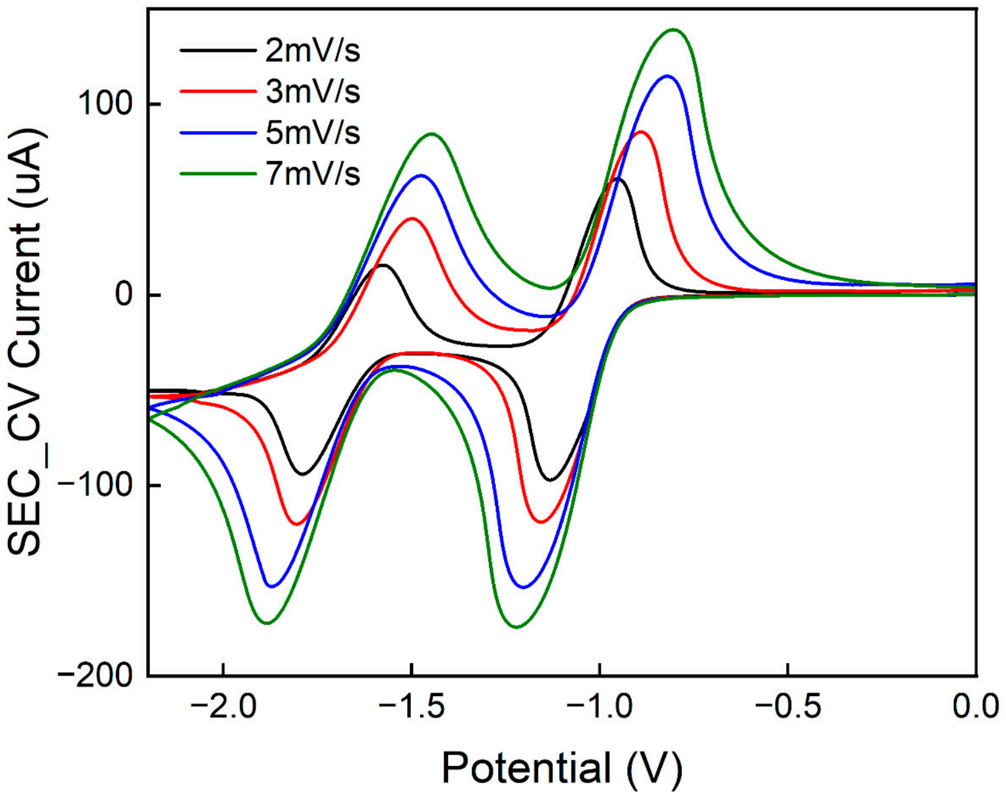

Figure S2: Experimental SEC-CV currents, recorded at different scan rates.

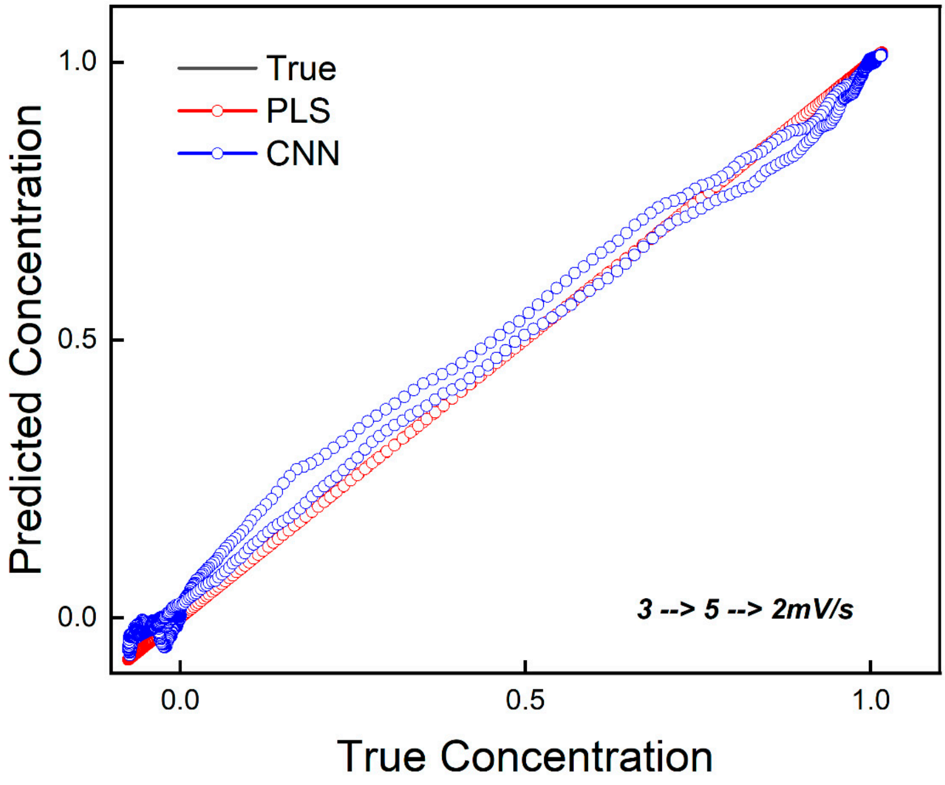

Figure S3: Parity plot of concentration (C1) values, comparing true values with single-SEC trained PLS and CNN model predictions.

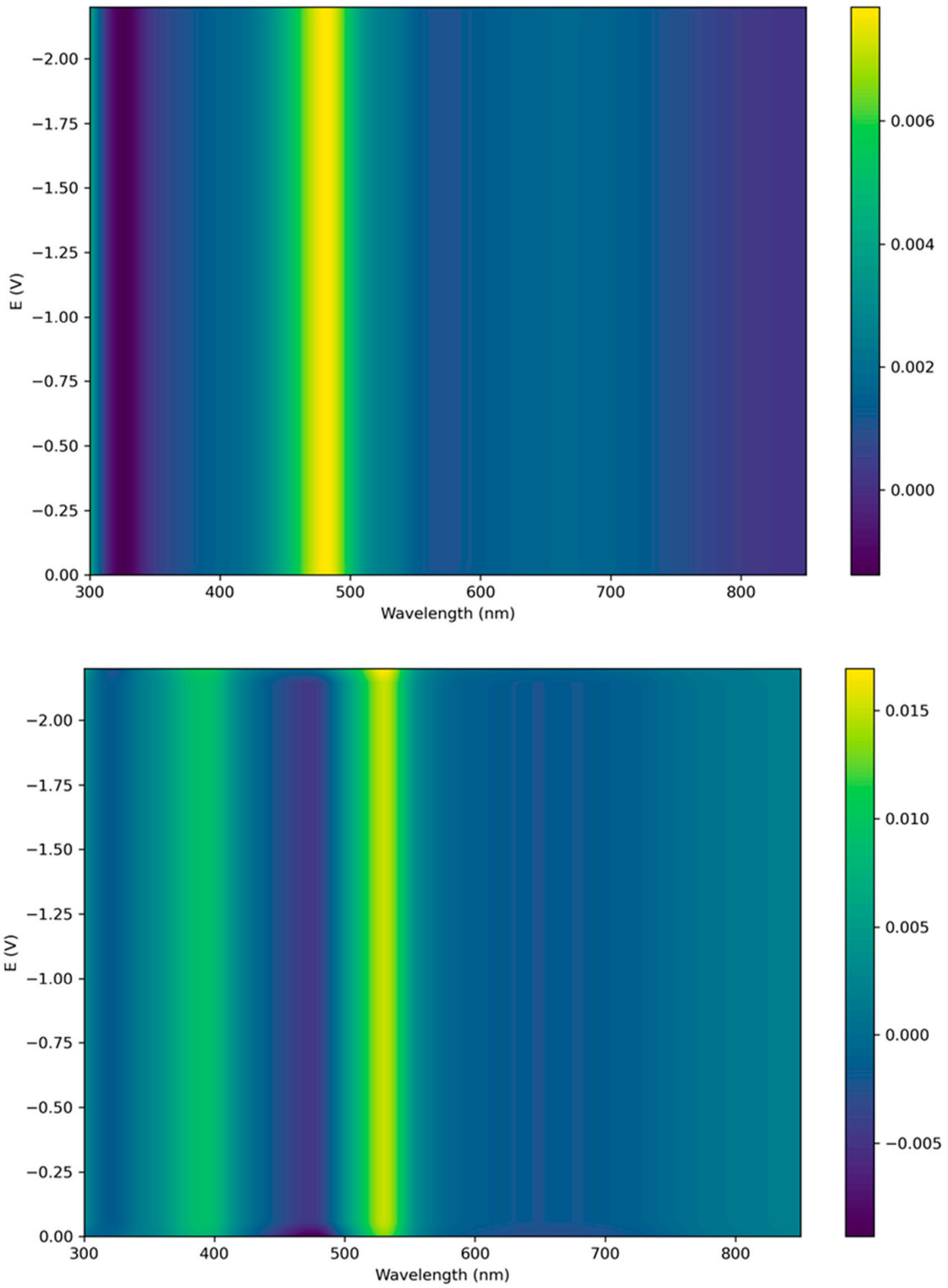

Figure S4: X loading of the first and second PLS components.

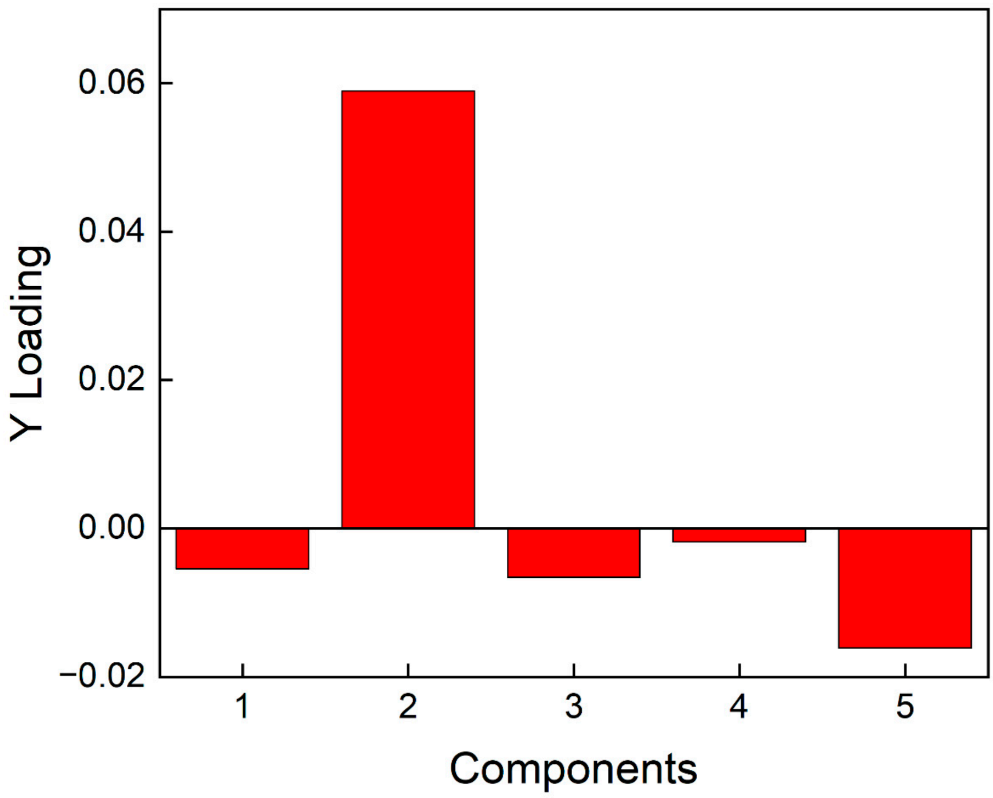

Figure S5: Y loading as a function of PLS components.

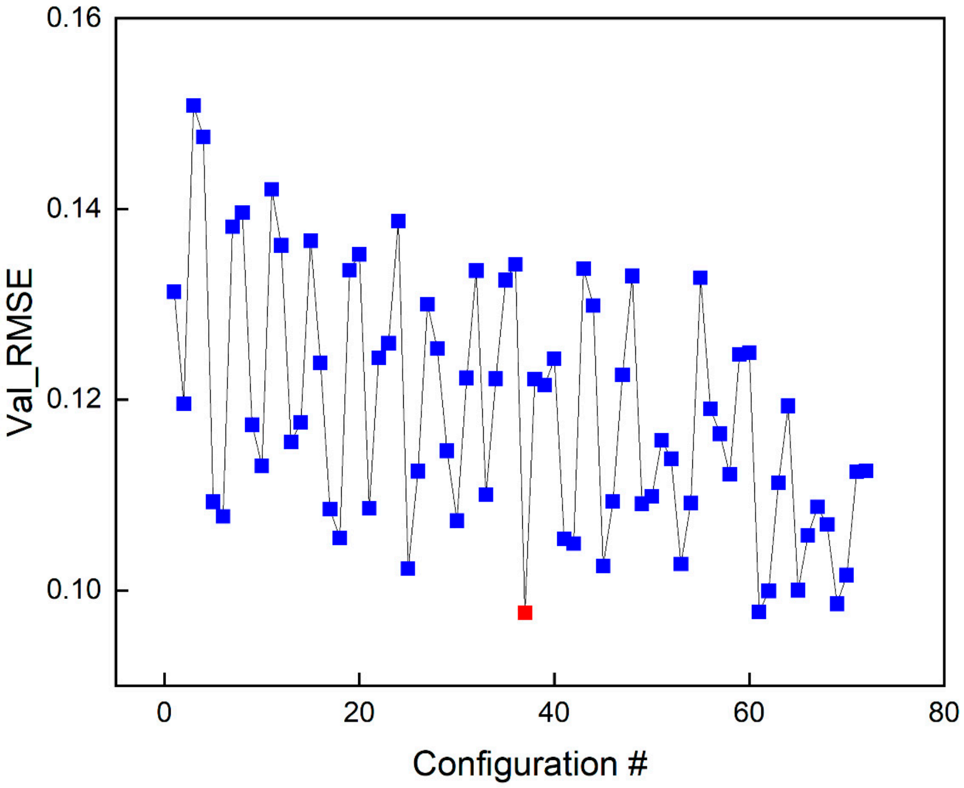

Figure S6: CNN hyperparameter sweep optimization based on validation RMSE.

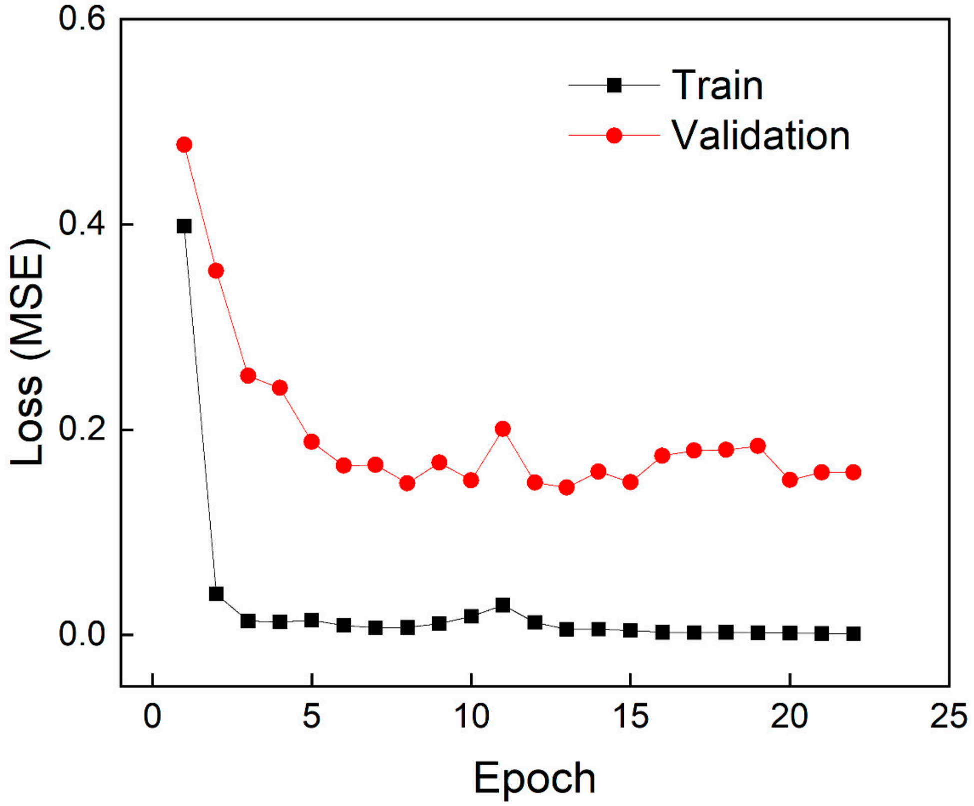

Figure S7: CNN training and validation losses as a function of epochs.

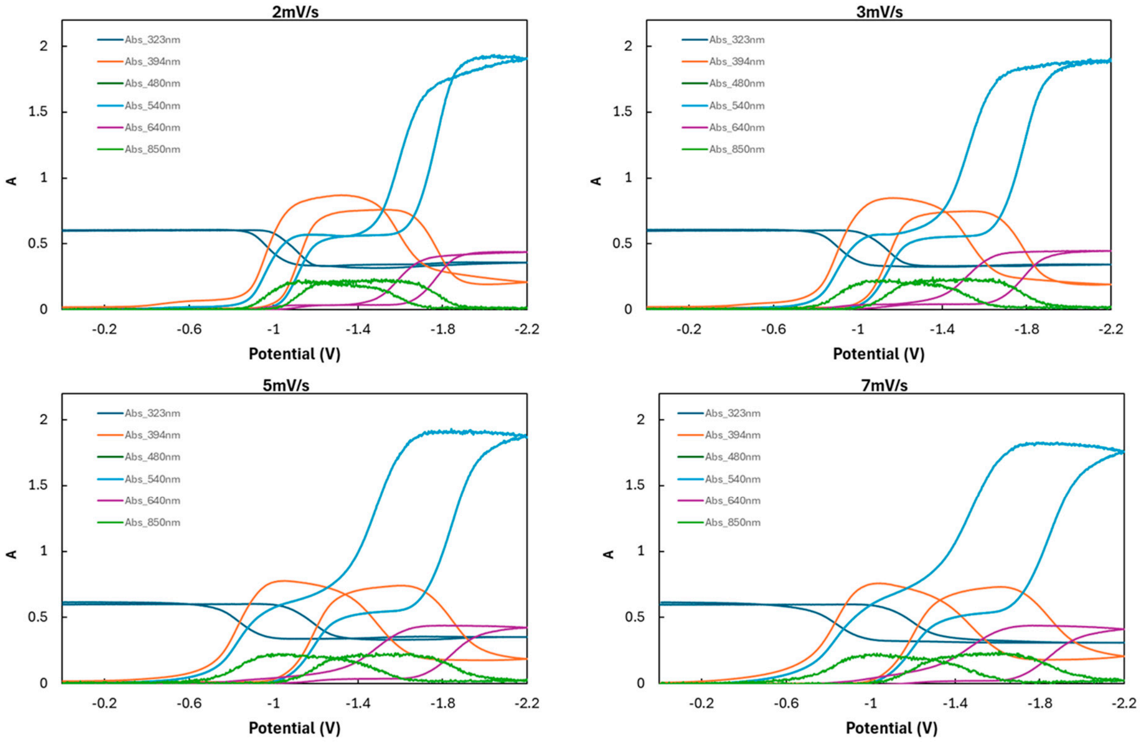

Figure S8: Change of absorbance at selected wavelengths as a function of applied potential across the four studied scan rates.

Table S1: Single-SEC performance for concentration.

Train scan_rate (V)	Validation scan_rate (V)	Test scan_rate (V)	PLS Concentration		CNN Concentration	
			Test_RMSE	Test_R2	Test_RMSE	Test_R2
0.005	0.003	0.002	0.0237	0.9977	0.0331	0.9956
0.007	0.003	0.002	0.0215	0.9981	0.0460	0.9914
0.003	0.005	0.002	0.0189	0.9985	0.0211	0.9982
0.007	0.005	0.002	0.0215	0.9981	0.0463	0.9913
0.003	0.007	0.002	0.0195	0.9984	0.0215	0.9981
0.005	0.007	0.002	0.0250	0.9975	0.0331	0.9956
0.005	0.002	0.003	0.0108	0.9995	0.0211	0.9981
0.007	0.002	0.003	0.0214	0.9981	0.0459	0.9911
0.002	0.005	0.003	0.0168	0.9988	0.0199	0.9983
0.007	0.005	0.003	0.0140	0.9992	0.0342	0.9951
0.002	0.007	0.003	0.0168	0.9988	0.0197	0.9984
0.005	0.007	0.003	0.0109	0.9995	0.0211	0.9981
0.003	0.002	0.005	0.0145	0.9991	0.0171	0.9987
0.007	0.002	0.005	0.0179	0.9986	0.0427	0.9921
0.002	0.003	0.005	0.0188	0.9985	0.0299	0.9961
0.007	0.003	0.005	0.0127	0.9993	0.0399	0.9931
0.002	0.007	0.005	0.0186	0.9985	0.0298	0.9961
0.003	0.007	0.005	0.0070	0.9998	0.0139	0.9992
0.003	0.002	0.007	0.0222	0.9978	0.0265	0.9969
0.005	0.002	0.007	0.0112	0.9995	0.0255	0.9972
0.002	0.003	0.007	0.0160	0.9989	0.0362	0.9942
0.005	0.003	0.007	0.0112	0.9995	0.0255	0.9972
0.002	0.005	0.007	0.0200	0.9982	0.0362	0.9943
0.003	0.005	0.007	0.0148	0.9990	0.0249	0.9973

Table S2: Single and pair SEC mapping performance for DCVA current.

Train scan_rate (V)	Validation scan_rate (V)	Test scan_rate (V)	PLS DCVA		CNN DCVA	
			Test_RMSE	Test_R2	Test_RMSE	Test_R2
0.005	0.003	0.002	0.1012	0.873	0.0698	0.939
0.007	0.003	0.002	0.1266	0.801	0.0887	0.902
0.003	0.005	0.002	0.0490	0.970	0.0451	0.975
0.007	0.005	0.002	0.1300	0.790	0.1101	0.849
0.003	0.007	0.002	0.0534	0.964	0.0389	0.981
0.005	0.007	0.002	0.1012	0.873	0.0850	0.910
0.005	0.002	0.003	0.0690	0.948	0.0426	0.980
0.007	0.002	0.003	0.0901	0.912	0.0662	0.953
0.002	0.005	0.003	0.0538	0.969	0.0415	0.981
0.007	0.005	0.003	0.0905	0.911	0.0745	0.940
0.002	0.007	0.003	0.0538	0.969	0.0438	0.979
0.005	0.007	0.003	0.0613	0.959	0.0492	0.974
0.003	0.002	0.005	0.0597	0.967	0.0620	0.964
0.007	0.002	0.005	0.0670	0.958	0.0776	0.944
0.002	0.003	0.005	0.0873	0.929	0.0924	0.920
0.007	0.003	0.005	0.0670	0.958	0.0645	0.961
0.002	0.007	0.005	0.0829	0.936	0.0946	0.916
0.003	0.007	0.005	0.0579	0.969	0.0580	0.968
0.003	0.002	0.007	0.0806	0.944	0.0760	0.950
0.005	0.002	0.007	0.0720	0.955	0.0973	0.918
0.002	0.003	0.007	0.1174	0.881	0.1153	0.885
0.005	0.003	0.007	0.0508	0.978	0.0499	0.978
0.002	0.005	0.007	0.1125	0.891	0.1145	0.887
0.003	0.005	0.007	0.0839	0.939	0.0764	0.950
0.005;0.007	0.003	0.002	0.1135	0.840	0.0756	0.929
0.003;0.007	0.005	0.002	0.0853	0.909	0.0428	0.977
0.003;0.005	0.007	0.002	0.0750	0.930	0.0484	0.971
0.005;0.007	0.002	0.003	0.0768	0.936	0.0489	0.974
0.002;0.007	0.005	0.003	0.0424	0.981	0.0220	0.995
0.002;0.005	0.007	0.003	0.0389	0.984	0.0268	0.992
0.003;0.007	0.002	0.005	0.0557	0.971	0.0526	0.974
0.002;0.007	0.003	0.005	0.0711	0.953	0.0450	0.981
0.002;0.003	0.007	0.005	0.0745	0.948	0.0539	0.973
0.003;0.005	0.002	0.007	0.0744	0.952	0.0549	0.974
0.002;0.005	0.003	0.007	0.0874	0.934	0.0453	0.982
0.002;0.003	0.005	0.007	0.0965	0.920	0.0691	0.959

Table S3: CNN hyperparameters sweep optimization and configurations.

CNN Configuration #	Filters	Kernel size	Dense units	Drop out	L2	Validation_RMSE
1	64	7	64	0	0	0.13133
2	64	7	64	0	0.0001	0.11957
3	64	7	64	0.2	0	0.15087
4	64	7	64	0.2	0.0001	0.14757
5	64	7	128	0	0	0.10929
6	64	7	128	0	0.0001	0.10777
7	64	7	128	0.2	0	0.13815
8	64	7	128	0.2	0.0001	0.13965
9	64	7	256	0	0	0.11738
10	64	7	256	0	0.0001	0.11305
11	64	7	256	0.2	0	0.14207
12	64	9	256	0.2	0.0001	0.13619
13	64	9	64	0	0	0.11556
14	64	9	64	0	0.0001	0.11762
15	64	9	64	0.2	0	0.13668
16	64	9	64	0.2	0.0001	0.12387
17	64	9	128	0	0	0.10853
18	64	9	128	0	0.0001	0.10552
19	64	9	128	0.2	0	0.13359
20	64	9	128	0.2	0.0001	0.13524
21	64	9	256	0	0	0.10860
22	64	9	256	0	0.0001	0.12439
23	64	9	256	0.2	0	0.12592
24	64	9	256	0.2	0.0001	0.13872
25	128	7	64	0	0	0.10228
26	128	7	64	0	0.0001	0.11249
27	128	7	64	0.2	0	0.13003
28	128	7	64	0.2	0.0001	0.12536
29	128	7	128	0	0	0.11464
30	128	7	128	0	0.0001	0.10730
31	128	7	128	0.2	0	0.12228
32	128	7	128	0.2	0.0001	0.13354
33	128	7	256	0	0	0.11003
34	128	7	256	0	0.0001	0.12220
35	128	7	256	0.2	0	0.13256
36	128	7	256	0.2	0.0001	0.13419
37	128	9	64	0	0	0.09765

38	128	9	64	0	0.0001	0.12216
39	128	9	64	0.2	0	0.12151
40	128	9	64	0.2	0.0001	0.12429
41	128	9	128	0	0	0.10540
42	128	9	128	0	0.0001	0.10492
43	128	9	128	0.2	0	0.13375
44	128	9	128	0.2	0.0001	0.12988
45	128	9	256	0	0	0.10256
46	128	9	256	0	0.0001	0.10932
47	128	9	256	0.2	0	0.12258
48	128	9	256	0.2	0.0001	0.13298
49	256	7	64	0	0	0.10909
50	256	7	64	0	0.0001	0.10987
51	256	7	64	0.2	0	0.11575
52	256	7	64	0.2	0.0001	0.11381
53	256	7	128	0	0	0.10278
54	256	7	128	0	0.0001	0.10918
55	256	7	128	0.2	0	0.13280
56	256	7	128	0.2	0.0001	0.11904
57	256	7	256	0	0	0.11643
58	256	7	256	0	0.0001	0.11217
59	256	7	256	0.2	0	0.12475
60	256	7	256	0.2	0.0001	0.12494
61	256	9	64	0	0	0.09775
62	256	9	64	0	0.0001	0.09994
63	256	9	64	0.2	0	0.11130
64	256	9	64	0.2	0.0001	0.11935
65	256	9	128	0	0	0.10001
66	256	9	128	0	0.0001	0.10574
67	256	9	128	0.2	0	0.10876
68	256	9	128	0.2	0.0001	0.10689
69	256	9	256	0	0	0.09860
70	256	9	256	0	0.0001	0.10160
71	256	9	256	0.2	0	0.11245
72	256	9	256	0.2	0.0001	0.11254

Table S4: PLS single and pair SEC mapping performance for CV current.

Train size	Train scan_rate (V)	Validation scan_rate (V)	Test scan_rate (V)	PLS CV	
				Test_RMSE	Test_R2
1	0.005	0.003	0.002	0.2108	0.5480
1	0.007	0.003	0.002	0.2738	0.2383
2	0.005;0.007	0.003	0.002	0.2210	0.5033
1	0.003	0.005	0.002	0.1049	0.8882
1	0.007	0.005	0.002	0.2738	0.2383
2	0.003;0.007	0.005	0.002	0.1786	0.6758
1	0.003	0.007	0.002	0.1067	0.8843
1	0.005	0.007	0.002	0.2108	0.5480
2	0.003;0.005	0.007	0.002	0.1560	0.7524
1	0.005	0.002	0.003	0.1119	0.8858
1	0.007	0.002	0.003	0.1723	0.7295
2	0.005;0.007	0.002	0.003	0.1386	0.8248
1	0.002	0.005	0.003	0.0972	0.9138
1	0.007	0.005	0.003	0.1709	0.7339
2	0.002;0.007	0.005	0.003	0.0552	0.9722
1	0.002	0.007	0.003	0.0934	0.9203
1	0.005	0.007	0.003	0.1119	0.8858
2	0.002;0.005	0.007	0.003	0.0440	0.9823
1	0.003	0.002	0.005	0.1107	0.9046
1	0.007	0.002	0.005	0.0848	0.9441
2	0.003;0.007	0.002	0.005	0.1116	0.9030
1	0.002	0.003	0.005	0.2090	0.6600
1	0.007	0.003	0.005	0.0830	0.9465
2	0.002;0.007	0.003	0.005	0.1135	0.8996
1	0.002	0.007	0.005	0.1961	0.7005
1	0.003	0.007	0.005	0.1297	0.8690
2	0.002;0.003	0.007	0.005	0.1561	0.8103
1	0.003	0.002	0.007	0.1545	0.8435
1	0.005	0.002	0.007	0.0779	0.9602
2	0.003;0.005	0.002	0.007	0.1526	0.8473
1	0.002	0.003	0.007	0.2154	0.6956
1	0.005	0.003	0.007	0.0779	0.9602
2	0.002;0.005	0.003	0.007	0.1630	0.8258
1	0.002	0.005	0.007	0.2342	0.6402
1	0.003	0.005	0.007	0.1545	0.8435
2	0.002;0.003	0.005	0.007	0.1772	0.7940

Table S5: CNN Single and pair SEC mapping performance for CV current.

Train size	Train scan_rate (V)	Validation scan_rate (V)	Test scan_rate (V)	CNN CV	
				Test_RMSE	Test_R2
1	0.005	0.003	0.002	0.1875	0.6423
1	0.007	0.003	0.002	0.1946	0.6151
2	0.005;0.007	0.003	0.002	0.2258	0.4819
1	0.003	0.005	0.002	0.1166	0.8618
1	0.007	0.005	0.002	0.2312	0.4569
2	0.003;0.007	0.005	0.002	0.0832	0.9297
1	0.003	0.007	0.002	0.1092	0.8788
1	0.005	0.007	0.002	0.2117	0.5444
2	0.003;0.005	0.007	0.002	0.1206	0.8521
1	0.005	0.002	0.003	0.1085	0.8926
1	0.007	0.002	0.003	0.1118	0.8861
2	0.005;0.007	0.002	0.003	0.1241	0.8596
1	0.002	0.005	0.003	0.0903	0.9256
1	0.007	0.005	0.003	0.1365	0.8303
2	0.002;0.007	0.005	0.003	0.0496	0.9776
1	0.002	0.007	0.003	0.0921	0.9226
1	0.005	0.007	0.003	0.1128	0.8839
2	0.002;0.005	0.007	0.003	0.0365	0.9878
1	0.003	0.002	0.005	0.1364	0.8552
1	0.007	0.002	0.005	0.1628	0.7940
2	0.003;0.007	0.002	0.005	0.1599	0.8013
1	0.002	0.003	0.005	0.1946	0.7053
1	0.007	0.003	0.005	0.0753	0.9559
2	0.002;0.007	0.003	0.005	0.1349	0.8586
1	0.002	0.007	0.005	0.1948	0.7047
1	0.003	0.007	0.005	0.1106	0.9048
2	0.002;0.003	0.007	0.005	0.1191	0.8895
1	0.003	0.002	0.007	0.1454	0.8613
1	0.005	0.002	0.007	0.0906	0.9461
2	0.003;0.005	0.002	0.007	0.1770	0.7946
1	0.002	0.003	0.007	0.2384	0.6271
1	0.005	0.003	0.007	0.0813	0.9566
2	0.002;0.005	0.003	0.007	0.1320	0.8858
1	0.002	0.005	0.007	0.2384	0.6271
1	0.003	0.005	0.007	0.1510	0.8505
2	0.002;0.003	0.005	0.007	0.1169	0.9103
